# Osteoblast‐derived extracellular vesicles exert osteoblastic and tumor‐suppressive functions via SERPINA3 and LCN2 in prostate cancer

**DOI:** 10.1002/1878-0261.13484

**Published:** 2023-08-04

**Authors:** Kagenori Ito, Tomofumi Yamamoto, Yusuke Hayashi, Shun Sato, Jun Nakayama, Fumihiko Urabe, Takeo Shimasaki, Eijiro Nakamura, Yoshiyuki Matui, Hiroyuki Fujimoto, Takahiro Kimura, Shin Egawa, Takahiro Ochiya, Yusuke Yamamoto

**Affiliations:** ^1^ Laboratory of Integrative Oncology National Cancer Center Research Institute Chuo‐ku Japan; ^2^ Department of Urology Jikei University School of Medicine Minato‐ku Japan; ^3^ Department of Molecular and Cellular Medicine Tokyo Medical University Shinjuku‐ku Japan; ^4^ Department of Pathology Jikei University School of Medicine Minato‐ku Japan; ^5^ Medical Research Institute Kanazawa Medical University Kahoku‐gun Japan; ^6^ Department of Urology and Retroperitoneal Surgery National Cancer Center Hospital Chuo‐ku Japan

**Keywords:** extracellular vesicles, LCN2, osteoblastic bone metastasis, prostate cancer, SERPINA3

## Abstract

Clinically, the osteolytic phenotype is rare in prostate cancer (PCa), and the prognosis is generally worse than that of the osteoblastic phenotype. Osteoblastic prostate cancer (BPCa) is a major type of bone metastasis. Several factors responsible for osteogenesis have been identified, but the molecular mechanism of osteoblastic bone metastasis in PCa is not fully understood. Here, we show the osteogenic and tumor‐suppressive roles of SERPINA3 and LCN2 in BPCa. In a co‐culture of osteoblasts (OBs) and BPCa cells, SERPINA3 and LCN2 were remarkably upregulated in BPCa via OB‐derived extracellular vesicles, while they were not in the co‐culture of OBs and osteolytic prostate cancer (LPCa) cells. In both the co‐culture system and mouse xenograft experiments with intracaudal injection, enhanced expression of SERPINA3 and LCN2 in PCa led to osteogenesis. Additionally, the addition of SERPINA3 and LCN2 to BPCa cells significantly suppressed the proliferative potential. Retrospective analysis also confirmed that high expression levels of SERPINA3 and LCN2 were significantly correlated with a better prognosis. Our results may partially explain how osteoblastic bone metastasis develops and why the prognosis for BPCa is relatively better than that for LPCa.

## Graphical abstract

The authors show that SERPINA3 and LCN2 have osteogenic and tumor‐suppressive roles at sites of bone metastases in osteoblastic prostate cancer. Osteolytic prostate cancer lacks this mechanism, leading to osteolytic bone metastases and poor survival. SERPINA3 and LCN2 may serve as diagnostic biomarkers to predict bone metastasis phenotype and survival.

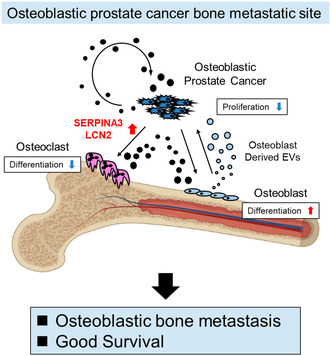

**Abbreviations**
AA: antibiotic antimycotic solution
BPCa: osteoblastic prostate cancer
CM: conditioned medium
CT: computed tomography
EVs: extracellular vesicles
IHC: immunohistochemistry
IVIS: *in vivo* imaging systems
LPCa: osteolytic prostate cancer
MRI: magnetic resonance imaging
NCC: National Cancer Center
OBs: osteoblasts
OCs: osteoclasts
OS: overall survival
PCa: prostate cancer
PFS: progression‐free survival
RNA‐seq: RNA sequencing
STR: short tandem repeat
TRAP: tartrate‐resistant acid phosphatase

## 1 Introduction

Prostate cancer (PCa) is the second leading cause of morbidity and sixth leading cause of mortality worldwide [1]. Despite the high morbidity, patients with localized PCa can achieve complete remission by operation or radiation. The 5‐year survival rate is almost 100% for localized PCa [2]. PCa often metastasizes mostly to bone and then relocates into other organs, such as lung and liver. Once metastasis occurs, the 5‐year survival rate decreases to 32.3% [2]. However, this rate of PCa is relatively higher than that of other cancers that frequently cause bone metastasis (breast 30%, kidney 15.3%, lung 7%, and liver 3.1%) [2]. PCa develops osteoblastic bone metastasis; other cancers largely develop osteolytic bone metastasis. This phenomenon could be explained by the relationship between the bone metastasis phenotype and prognosis.

The phenotype of bone metastasis varies among cancer types. Bone metastases are osteolytic in most types of cancers; for example, only 20% of bone metastases of breast cancer and several lung cancers are osteoblastic; others are osteolytic bone metastases. However, 99% of bone metastases in PCa are osteoblastic. PCa seems to have a specific process to induce osteogenesis rather than osteolysis [3]. Clinically, Shimasaki et al. [4] reported that the population with osteolytic bone metastasis of PCa had a worse prognosis than that of the osteoblastic phenotype. Laorden et al. reported that C‐telopeptides of type 1 collagen (CICP) and bone‐specific ALP (BALP) were osteoblastic markers, indicating good prognosis in PCa [5]. Based on these data, the osteoblastic bone metastatic phenotype of PCa may contribute to good survival.

Bone matrix is mainly formed by calcium, phosphate, and collagen. In the bone matrix, there are several types of cells, such as osteocytes, osteoblasts (OBs), osteogenic cells, and osteoclasts (OCs). Among them, OBs and OCs degrade and produce the matrix to renew the bone, maintaining the balance of bone metabolism [6]. Once cancer cells invade beside the bone matrix, cancer cells affect this bone metabolism and disrupt the balance. In the case of PCa, an increase in bone matrix is observed in osteoblastic prostate cancer (BPCa), and in contrast, a decrease in bone matrix is found in osteolytic prostate cancer (LPCa) [7]. Cancer cells release many humoral factors, such as proteins, extracellular vesicles (EVs), and cytokines, which might influence the OB and OC balance. Many humoral factors, such as BMP2, TGFβ, IGF1, IGFBP3, PDGF, FGF, VEGF, WNT, ET1, PSA, uPA, and MDA‐BF‐1, released by PCa are known to play a role in osteogenesis [8]. However, the key factors that create osteoblastic bone metastasis are still unclear.

In this study, to clarify the relationship between osteoblastic bone metastasis in PCa and the relatively better prognosis compared with osteolytic bone metastasis, we evaluated the effect of intercellular communication among BPCa cells, LPCa cells, OBs, and OCs in a horizontal co‐culture system. We found that the interaction between BPCa cells and OBs specifically induced SERPINA3 and LCN2 expression in BPCa via OB‐derived EVs. Gain‐of‐function experiments of SERPINA3 and LCN2 in the horizontal co‐culture system and NICO‐1 and mouse xenograft experiments showed osteogenic and tumor‐suppressive roles in PCa. Using publicly available large‐scale datasets and plasma from PCa patients, we also showed the clinical relevance of SERPINA3 and LCN2 in PCa.

## 2 Materials and methods

### 2.1 Cell lines and cell culture

The human PCa cell lines DU145 (RRID:CVCL_0105; ATCC, Manassas, VA, USA) and VCaP (RRID:CVCL_2235; ATCC) were cultured in DMEM (Nacalai Tesque, Inc., Kyoto, Japan) with 10% heat‐inactivated FBS (ATCC) and 1% antibiotic‐antimycotic solution (AA; Thermo Fisher Scientific, Waltham, MA, USA) at 37 °C. The human PCa cell lines 22Rv1 (RRID:CVCL_1045; ATCC) and PC‐3M‐luc‐C6 (RRID:CVCL_D577; Xenogen, Alameda, CA, USA) were cultured in RPMI 1640 medium (Nacalai Tesque, Inc.) with 10% heat‐inactivated FBS and 1% AA at 37 °C. The human PCa cell lines C4 (RRID:CVCL_4783; ATCC), C4‐2 (RRID:CVCL_4782; ATCC), and C4‐2B (RRID:CVCL_4784; ATCC) were cultured in DMEM/F12 (4 : 1) (Nacalai Tesque, Inc.) with 0.100 μg·mL^−1^ insulin, 275 ng·mL^−1^ triiodothyronine, 88.6 ng·mL^−1^ apo‐transferrin, 4.9 ng·mL^−1^ d–biotin, 251.8 ng·mL^−1^ adenine (T‐medium), 10% heat‐inactivated FBS, and 1% AA at 37 °C. All components of T‐medium were combined, mixed well, and sterilized using a 0.22‐μm filter, and 5.7 mL was aseptically dispensed into sterile tubes. The murine monocytic cell line RAW264.7 (RRID:CVCL_0493; ATCC), human osteosarcoma cell lines MG63 (RRID:CVCL_0426, ATCC) and Saos2 (RRID:CVCL_0548; ATCC), and murine pre‐OB cell line MC3T3‐E1 subclone 4 (RRID:CVCL_5440; ATCC) were cultured in αMEM without ascorbic acid (Thermo Fisher Scientific) with 10% heat‐inactivated FBS and 1% AA at 37 °C. RAW264.7 cells were used in passages 6–10 from purchase. For routine maintenance, each cell line was grown as a monolayer at 37 °C with 5% carbon dioxide and 95% relative humidity. C4, C4‐2, C4‐2B, 22Rv1, MC3T3‐E1 subclone4, RAW264.7, and VCaP cell lines were purchased from ATCC in the past 3 years. DU145, PC‐3M‐luc‐C6, MG63, and Saos2 cell lines were confirmed to be free of cross‐contamination with human cultured cells using Promega's (Madison, WI, USA) short tandem repeat (STR) analysis system and the STR database constructed from the Japanese Collection of Research Bioresources (JCRB) cell bank in the past 3 years. All cell lines were checked as mycoplasma free (MycoAlert Mycoplasma Detection Kit; Lonza, Basel, Switzerland). We used DU145 [9], 22Rv1 [10], and PC‐3M as LPCa; C4‐2 [11], C4‐2B [12], and VCaP [13] as BPCa, whose bone metastasis phenotypes are shown in sited papers.

### 2.2 Horizontal co‐culture

Each PCa cell line (DU145; 6.0 × 10^3^ cells, 22Rv1; 3.0 × 10^4^ cells, C4‐2; 1.5 × 10^4^ cells, C4‐2B; 1.5 × 10^4^ cells, PC‐3M; 6.0 × 10^3^ cells, and VCaP; 2.0 × 10^5^) was co‐cultured in a horizontal co‐culture system (NICO‐1; Ginrei Lab, Kanazawa, Japan) with and without murine monocytic cells (pre‐OC, RAW264.7; 7.5 × 10^3^ cells) to evaluate the effect of humoral factors secreted from RAW264.7 cells on PCa cells and humoral factors secreted from PCa cells on RAW264.7 cells. Each PCa cell line was also co‐cultured with and without OB‐like cells (MG63; 5.0 × 10^3^ cells, Saos2; 2.0 × 10^4^ cells, and MC3T3; 3.0 × 10^4^ cells). Filters of 0.03 and 0.6 μm were inserted between wells as previously described [14]. αMEM with ascorbic acid (Thermo Fisher Scientific) with 10% heat‐inactivated FBS was used for the co‐culture method.

### 2.3 Boyden co‐culture

Each PCa cell line (PC‐3M; 6.0 × 10^3^ cells and C4‐2B; 1.5 × 10^4^ cells) was co‐cultured in the upper Boyden chamber of a 12‐well plate (vertical co‐culture) with murine monocytic cells (RAW264.7; 1.0 × 10^4^ cells) or OB‐like cells (MG63; 6666 cells, Saos2; 2.0 × 10^5^ cells, and MC3T3‐E1; 2.0 × 10^5^ cells) in the lower chamber of a 12‐well plate to evaluate the effect of humoral factors secreted from PCa cells on RAW264.7 and OB‐like cells. αMEM with ascorbic acid (Thermo Fisher Scientific) with 10% heat‐inactivated FBS was used for the co‐culture method. For EV inhibition, GW4869 (5 or 10 μm; Selleck Chemicals, Houston, TX, USA) was applied to culture medium of MG63 and C4‐2B.

### 2.4 Osteoclast differentiation

RAW264.7 cells were seeded in NICO‐1 at a density of 7.5 × 10^3^ cells per well or seeded in 96‐well plates at a density of 4.0 × 10^3^ cells per well. Recombinant mouse sRANKL (GST‐RANKL; Oriental Yeast Co., Tokyo, Japan) was administered at a concentration of 50 ng·mL^−1^ along with seeding of RAW264.7 cells. On Day 5, the cells were stained with tartrate‐resistant acid phosphatase (TRAP) using a TRAP Staining Kit (Cosmo Bio, Tokyo, Japan). TRAP‐positive multinuclear cells containing three or more nuclei were counted as osteoclasts under a light microscope.

### 2.5 qRT–PCR

Total RNA was isolated from the cell pellets using an RNeasy Mini Kit (Qiagen, Hilden, Germany). RNA was converted to first‐strand cDNA using the High‐Capacity cDNA Reverse Transcription Kit (Applied Biosystems, Waltham, MA, USA). Real‐time PCR analyses were performed using Platinum™ SYBR™ Green qPCR Super Mix‐UDG (Thermo Fisher Scientific). The expression of mRNA was normalized to β‐actin or B2‐microglobulin. Raw qRT‐PCR data from RAW246.7 cells were normalized to B2 microglobulin. Others were normalized to β‐actin. All primer sequences are shown in Table S1.

### 2.6 Whole RNA sequencing

RNA was isolated by an RNeasy Mini Kit (Qiagen) from each cell lysate. RNA sequencing was performed using DNBSEQ‐G400 (MGI Tech, Shenzhen, China) by Genewiz (Saitama, Japan). Expression levels for each gene were quantified from the sequencing data by kallisto (ver. 0.46.0) [15]. Afterward, the data were summarized using the tximport package (ver. 1.18.0) of r software (ver. 3.6.3), and scaled TPM counts were used for further analysis. Genes with low read coverage (maximum read count: < 100 reads) were excluded.

### 2.7 Expression vector constructs

SERPINA3 (pLV[Exp]‐Hygro‐EF1A > hSERPINA3, ID: VB210404‐1086jxf), LCN2 (pLV[Exp]‐Bsd‐CBh > hLCN2, ID: VB210622‐1321dgd), and empty (pLV[Exp]‐Puro‐EF1A > ORF, ID: VB900122‐0484ubz) expression vectors were produced by Vector Builder (Chicago, IL, USA). The luciferase (pLenti‐CMV‐luc‐puro) expression vector was kindly provided by Dr Yosuke Tanaka (the National Cancer Center (NCC) Research Institute). The Venus (pLenti‐EF1‐Venus) expression vector was kindly provided by Ms Yuka Kuroiwa (NCC Research Institute).

### 2.8 Lentiviral packaging and infection

HEK293T cells were seeded in 10‐cm culture dishes at a density of 2.0 × 10^6^ and transfected with 6 μg of lentiviral plasmids mixed with 3 μg VSVG, 3 μg LP1, 3 μg LP2, and 60 μL of Lipofectamine 2000 reagent (Thermo Fisher Scientific). Six hours after transfection, the culture supernatant was replaced with DMEM. Forty‐eight hours after medium replacement, the culture supernatant was harvested and centrifuged and then filtered through a 0.45 μm filter to remove cell debris. PC3M‐luc‐SERPINA3, PC3M‐luc‐LCN2, PC3M‐luc‐Venus, C4‐2B‐luc, C4‐2B‐luc‐SERPINA3, C4‐2B‐luc‐LCN2, and C4‐2B‐luc‐Venus cells were established by lentiviral infection of the target genes via administration of plasmid‐infected HEK293T‐conditioned medium (CM). SERPINA3‐overexpressing cells were selected with 150 μg·mL^−1^ hygromycin B (Invitrogen, Waitham, MA, USA) for 5 days. LCN2‐overexpressing cells were selected with 7.5–10 μg·mL^−1^ blasticidin S HCl (Thermo Fisher) for 5 days. Luciferase‐overexpressing cells were selected with 1 μg·mL^−1^ puromycin (Thermo Fisher) for 2 days.

### 2.9 Patient plasma samples

The collection and usage of human plasma from PCa patients (*n* = 39) by the NCC were approved by National Cancer Center Institutional Review Board (No. 2020‐078). Plasma was collected from NCC patients at outpatient visits, which were undertaken with the understanding and written consent of each subject, aliquoted and stored at −80 °C until use, and freeze–thawing was avoided thereafter (January 2010 to December 2020). This study's methodologies conformed to the standards set by the Declaration of Helsinki.

### 2.10 Caspase activity assay

Caspase activity was determined by using the Caspase‐Glo 3/7 Assay System (Promega) according to the manufacturer's instructions. Luminescence was measured by using a SpectraMax iD3 plate reader was made by Molecular Devices (San Jose, CA, USA).

### 2.11 Invasion assay, wound healing assay, and colony formation assay

All assays were performed by C4‐2B cell lines (C4‐2B, C4‐2B‐SERPINA3, C4‐2B‐LCN2, and C4‐2B‐SERPINA3‐LCN2). Images were acquired with a BZ‐X700 microscope (Keyence Corporation, Osaka, Japan) and analyzed using imagej software. Invasion assay was performed by seeding cells (1.0 × 10^5^ cells/well) on the 48‐well‐sized collagen‐coated upper chamber of 8‐μm‐pore‐size transwell inserts (Corning, Corning, NY, USA) with serum‐free DMEM medium (Thermo Fisher Scientific). 20% FBS added DMEM medium was added in lower chamber. After incubation for 24 h, migrated cells at the lower surface of the membrane were fixed with 4% paraformaldehyde–PBS (Fujifilm Wako Pure Chemical Corporation, Osaka, Japan) and stained with 0.005% crystal violet solution (Fujifilm Wako Pure Chemical Corporation). Then, margin liquid and cells on the upper surface of the membrane were removed with a cotton swab. Wound healing assay was performed by seeding cells (2.0 × 10^6^ cells/well) on the 35 mm dish. After incubation for 24 h until cell attaches to plate as 100% confluent, wounds were generated using a 200 μL micropipette tip. After incubation for 18 h, analysis was performed. Colony formation assay was performed by DMEM culture medium containing 0.3% agarose (Cytiva, Tokyo, Japan) with cells (1.0 × 10^4^ cells/well) over a bottom layer of 0.6% agarose in DMEM culture medium was plated in each well of a 6‐well plate and cultured for 3 weeks. Colonies were fixed with 4% paraformaldehyde–PBS for 10 min and stained with 0.005% crystal violet solution for 10 min. After removing the overdyed region with Milli‐Q water, colony numbers were calculated.

### 2.12 ELISA

SERPINA3 and LCN2 concentrations were determined by ELISA using the Human Alpha 1 Antichymotrypsin ELISA Kit (ab157706; Abcam, Cambridge, UK) and Human Lipocalin‐2 ELISA Kit (ab119600; Abcam), respectively, according to the manufacturer's instructions. Absorbance was measured by using a SpectraMax iD3 plate reader.

### 2.13 Preparation of conditioned medium and EVs

The cells were washed with phosphate‐buffered saline without calcium chloride and magnesium chloride (PBS−), and the culture medium was replaced with advanced DMEM (Thermo Fisher Scientific) for DU145 and MG63 cells, advanced RPMI 1640 medium (Thermo Fisher Scientific) for 22Rv1 cells, and advanced DMEM (Thermo Fisher Scientific)/advanced DMEM/F12 (Thermo Fisher Scientific) (3 : 2) for C4‐2 and C4‐2B cells. Each advanced medium contained 1% AA and 2 mm l‐glutamine. EVs from the CM of cells were isolated by a differential ultracentrifugation protocol, as we previously reported [16]. Briefly, the CM was centrifuged at 2000 ***g*** for 10 min to remove contaminating cells. The resulting supernatants were then transferred to fresh tubes and filtered through a 0.22 μm filter (Millipore, Burlington, MA, USA). The filtered CM was centrifuged for 70 min at 110 000 ***g*** to pellet the enriched EVs (Beckman Coulter, Rea, CA, USA). The pellets were washed with 11 mL of PBS and ultracentrifuged at 110 000 ***g*** for another 70 min. The EV pellets were stored in the refrigerator at 4 °C until use. The fraction containing the EVs was measured for its protein content using the Quant‐iTTM Protein Assay with Qubit2.0 Fluorometer (Invitrogen).

### 2.14 PKH67‐labeled EV transfer

Purified EVs derived from MG63 cells were labeled with the PKH67 Green Fluorescent Labeling Kit (Sigma‐Aldrich, St. Louis, MO, USA). EVs were incubated with 2 μm PKH67 for 5 min, washed four times through a 100‐kD filter (Microcon YM‐100; Millipore) to remove excess dye, and incubated with C4‐2B cells at 37 °C.

### 2.15 Nanoparticle tracking analysis

Extracellular vesicles were resuspended in PBS and further diluted for analysis in a Nanosiht is made by Quantum Design (San Diego, CA, USA) LM10‐HS system according to the manufacturer's protocol.

### 2.16 Bone metastasis assay *in vivo* and isolation of tumor cells from bone metastatic lesions

NOD SCID (NOD.CB17‐Prkdc^scid^/J) mice were purchased from The Jackson Laboratory (Bar Harbor, ME, USA) and housed in the specific pathogen‐free area. The experiments were approved by NCC (T19‐003‐M02) and carried out in consideration of the “Guideline for Animal Experiments in National Cancer Center” and the 3Rs stipulated by the Animal Welfare Management Act. The caudal artery injection procedure in mice has been previously reported [17]. C4‐2B‐luc (1.0 × 10^6^ cells/100 μL of PBS−) was injected into the caudal artery of 5‐week‐old male SCID mice. Tumors were monitored by IVIS Lumina II (PerkinElmer, Waitham, MA, USA) every week until bone metastatic tumors appeared in the femur or tibia. After the mouse was sacrificed, the femur and tibia were isolated. Both edges of the bones were removed. The bone marrow cavity was washed with PBS through a 23‐gauge injection needle in a 6‐cm dish several times. The legs of the transplanted mice were cut into small pieces with sterilized forceps in the same 6‐cm dishes with C4‐2B culture medium. The medium was replaced every day until cancer cells invaded the bone tissues attached to the bottom of the dish. Debris of bone and muscle were removed when cancer cells proliferated in the dishes. Cancer cells were selected with 1 μg·mL^−1^ puromycin until no floating dead cells were observed.

PC‐3M (1.0 × 10^6^ cells/100 μL of PBS−) was injected into mice as shown above. After 5 weeks, the mouse was sacrificed, and both legs were separated from the pelvis, fixed in 4% paraformaldehyde PBS−, and embedded in paraffin.

### 2.17 *In vivo* bioluminescence imaging

Beetle luciferin and potassium salt (Promega) were dissolved in PBS to a final concentration of 15 mg·mL^−1^ and then filtered using a 0.22 μm filter. The luciferin solution was injected at 10 μL·g^−1^ of mouse weight into each mouse intraperitoneally. After 10 min, bioluminescence was imaged using an IVIS Lumina II system (PerkinElmer) [18].

### 2.18 Patient tissue samples

The collection and usage of human tissue from PCa patients (*n* = 5) by the Jikei University, Japan, were approved by the Jikei University Institutional Review Board (No 21‐102(5680)). Prostate biopsy tissue taken at the initial diagnosis and lumbar spine tissue taken after multiple osteoblastic bone metastasis formation following treatment failure were each from the same patient and were undertaken with the understanding and written consent of each subject (January 2010 to December 2022). Tissues were fixed with 10% neutral buffered formalin and embedded in paraffin. This study's methodologies conformed to the standards set by the Declaration of Helsinki.

### 2.19 Immunohistochemical staining

Human samples were fixed with 4% paraformaldehyde (30525‐89‐4; Fujifilm, Osaka, Japan) in PBS and embedded in paraffin. The paraffin‐embedded samples were baked for 1 h at 60 °C before proceeding with the following steps. The paraffin sections were deparaffinized and rehydrated in xylene, a graded ethanol series that decreased stepwise from 100% to 50%, and distilled water. For immunohistochemistry (IHC) analysis, antigen retrieval was performed by proteinase K (S3020; Agilent, Dako, Santa Clara, CA, USA) at room temperature for 3 min. Endogenous peroxidase activity was blocked with 3% H_2_O_2_ in ultrapure water for 30 min. Then, these sections were incubated in 2.5% normal horse serum (Vector Laboratories, Newark, CA, USA) for 20 min at room temperature. The specimens were incubated with primary antibodies against SERPINA3 (HPA000893; Sigma‐Aldrich Co.) by 1 : 5000 dilution and LCN2 (HPA002695; Sigma‐Aldrich Co.) by 1 : 1000 dilution at 4 °C overnight. Then, the sections were incubated with the secondary antibody solutions for 30 min at room temperature. The sections were stained with an ImmPACT^®^ DAB EqV substrate kit (Vector Laboratories) followed by staining with hematoxylin, dehydration, and mounting. Images were acquired with a BZ‐X700 microscope (Keyence Corporation) and analyzed using image analysis. All rights reserved. No reuse is allowed without permission.

Mouse samples were fixed with 4% paraformaldehyde in PBS. Decalcification was performed by shaking the samples at 25 °C for 4 days using 1% ZnSO_4_ (Fujifilm) added to 10% EDTA2Na (20251; Muto Pure Chemicals, Tokyo, Japan) as the decalcification solution. Paraffin embedding was performed in an automatic machine (0403‐00; Sakura seiki, Nagano, Japan). Paraffin‐embedded samples were then sliced with a microtome (TRAP stain: 3 μm, ALP stain: 6 μm) and affixed to microscope slides. Paraffin sections were deparaffinized as described above. TRAP and ALP staining was performed using the TRAP/ALP Stain Kit (294‐67001; Fujifilm).

### 2.20 Statistical analysis

The data presented in the bar graphs are the means ± SDs of at least three independent experiments. The statistical analyses were performed with Student's *t*‐test, which was used to evaluate the co‐culture effect. Analysis of variance (ANOVA) was used for multiple comparisons, followed by Tukey's or Dunnett's multiple comparison test. Survival curves were drawn with the Kaplan–Meier method and analyzed with the log‐rank test. Significance was defined as *P* < 0.05. The statistical software used was prism version 8 (GraphPad Software, Inc.).

## 3 Results

### 3.1 LPCa is more progressive than BPCa

**Fig. 1:**
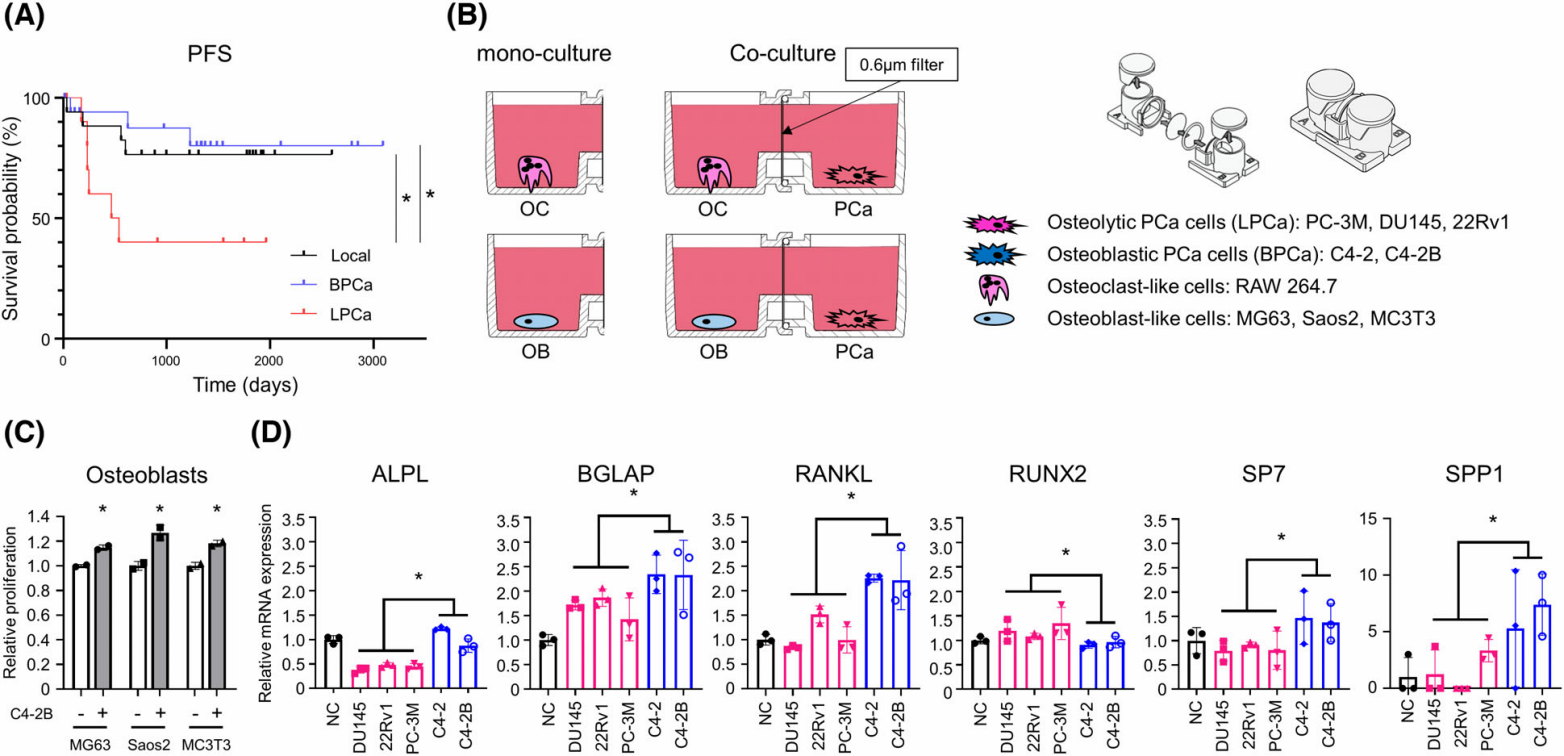
Horizontal co‐culture of OBs with LPCa or BPCa cells. (A) PSA progression‐free survival in patients with local PCa, BPCa, and LPCa. *P < 0.05; based on log‐rank test. (B) Schema of the horizontal co‐culture system using NICO‐1. A 0.6 μm filter was set between two wells to observe an interaction of two cell types. Mono‐culture was prepared as a control. (C) Proliferation of OBs with and without BPCa cells in the horizontal co‐culture system. *n* = 3, *P < 0.05; based on Student's *t* test. (D) Expression of OB differentiation marker genes in OBs measured by qRT–PCR with and without PCa cells (LPCa and BPCa). *n* = 3, *P < 0.05; based on Student's *t* test. All error bars indicate SD.

Osteolytic prostate cancer was reported to be a more progressive cancer than BPCa [4, 5]. To validate these reports, we retrospectively analyzed data from PCa patients at the National Cancer Institute, Japan. Fifty‐two patients were assigned to either the no bone metastasis cancer (local) group (*n* = 21), osteoblastic bone metastasis (BPCa) group (*n* = 19), or osteolytic bone metastasis group (LPCa) (*n* = 12) by bone imaging examination (CT, MRI, and bone scintigraphy). PSA progression free survival (PFS) was significantly shorter in LPCa group than in BPCa and local group (*P* = 0.0229 and *P* = 0.0477, Fig. 1A). Although overall survival (OS) showed no difference (*P* = 0.4960, Fig. S1A), which due to the short observation and relatively long survival of PCa. The baseline demographic characteristics were determined, and largely, the bone metastatic PCa group (osteoblastic and osteolytic) exhibited an aggressive phenotype compared with local PCa group (Table S2 and Fig. S1B). However, no significant difference in characteristics was observed between the osteoblastic and osteolytic bone metastasis groups (Table S2). These classifications, which are commonly used in clinical practice, cannot be used to delineate BPCa versus LPCa. Thus, consistent with previous reports, our data also showed a poorer prognosis in LPCa than BPCa. A new clinical classification that distinguishes between BPCa and LPCa is useful to predict survival of PCa.

### 3.2 OBs proliferate and differentiate in horizontal co‐culture with BPCa cells

To investigate molecular differences between LPCa and BPCa, we focused on the relationship between bone resident cells and cancer cells. Humoral factors and EVs from PCa are known to affect the proliferation and differentiation of OCs and OBs [19]. To further examine intercellular communication, we used a horizontal co‐culture system instead of a vertical co‐culture system because this system can promote mutual interaction between two cell types [20].

First, to determine the effect of PCa on pre‐OC proliferation and differentiation, we cultured RAW264.7 cells with either LPCa or BPCa cells with sRANKL (50 ng·mL^−1^) (Fig. 1B). RAW264.7 cells are known to differentiate in OCs by RANKL medication [21]. Co‐culture with both DU145 (LPCa) cells and C4‐2B (BPCa) cells slightly inhibited the growth of RAW264.7 cells compared with mono‐culture (Fig. S2A). On the other hand, qRT–PCR analysis and microscopic observation showed OC differentiation by co‐culture with DU145 and C4‐2B cells compared with mono‐culture (Fig. S2B,C). In particular, C4‐2B cells promoted clear differentiation of RAW264.7 cells. These results suggested that both BPCa and LPCa cells suppress proliferation and induce differentiation in OCs. Thus, because no obvious difference in OCs was observed between LPCa and BPCa cells, factors determining the phenotype of bone metastases could not be explained by their interaction with OCs.

Next, to determine the effect of PCa cells on OB proliferation and differentiation, we cultured OB‐like cells such as MG63, Saos2, and MC3T3 with either LPCa (DU145, 22Rv1, and PC‐3M) or BPCa (C4‐2 and C4‐2B) cells (Fig. 1B). The growth of all OB‐like cells was significantly increased by co‐culture with C4‐2B cells (Fig. 1C). qRT–PCR analysis showed that OB‐like cells co‐cultured with BPCa cells had significantly increased OB differentiation markers, except RUNX2, compared with co‐culture with LPCa cells (Fig. 1D). These results suggested that humoral factors from BPCa cells, but not LPCa cells, selectively increased OB proliferation and differentiation. Thus, we speculated that the phenotypic differences in bone metastasis between BPCa and LPCa rely mainly on the OB interaction but not the OC interaction.

### 3.3 SERPINA3 and LCN2 were selectively overexpressed in BPCa cells co‐cultured with OB‐like cells

**Fig. 2:**
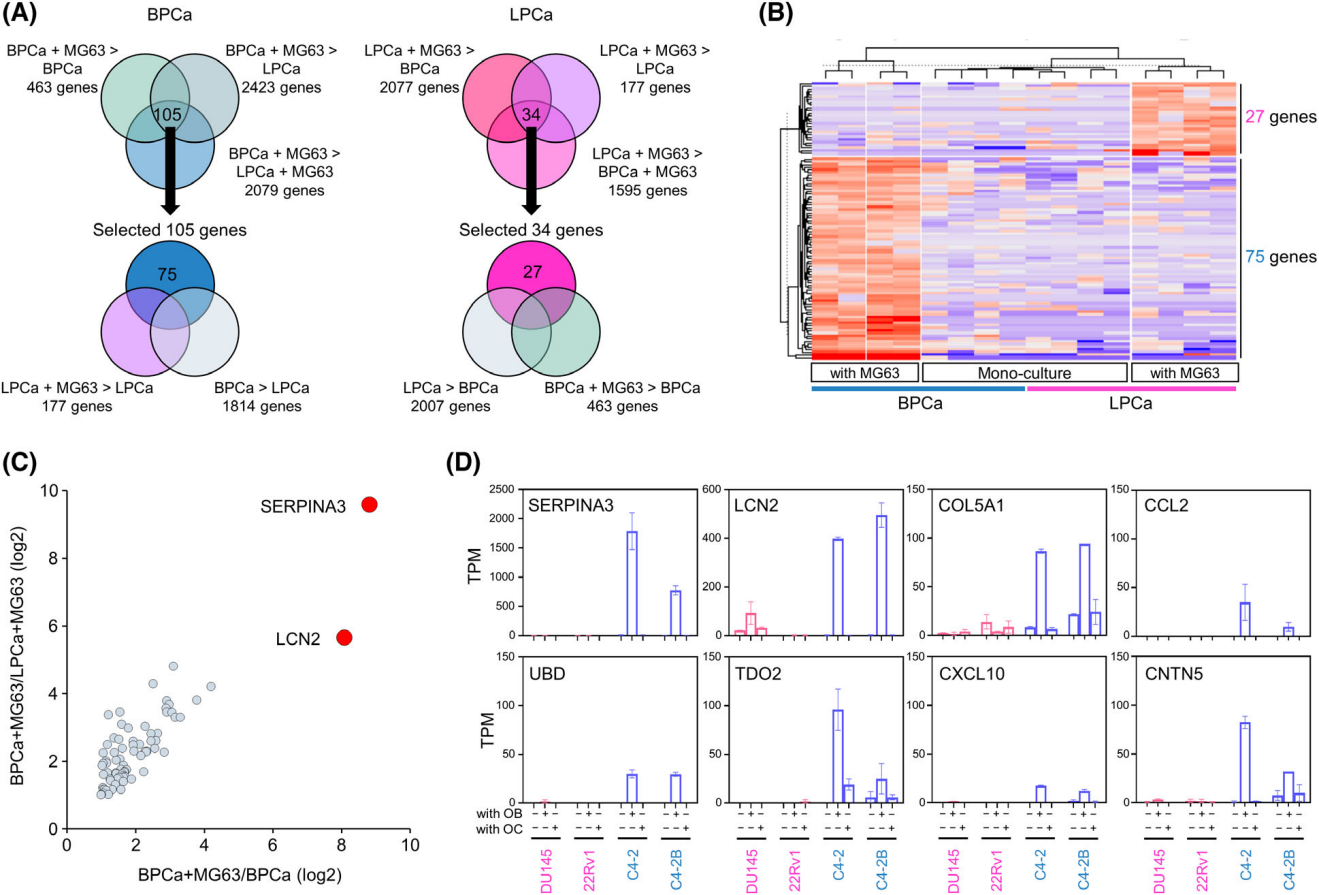
Specific upregulation of SERPINA3 and LCN2 in BPCa cells by horizontal co‐culture with MG63 cells. (A) Venn diagrams selecting 75 genes in BPCa (C4‐2 and C4‐2B) and 27 genes in LPCa (DU145 and 22Rv1) upregulated by horizontal co‐culture with MG63 cells (*n* = 3, fold change > 2‐fold and *P* < 0.05). (B) A heatmap indicating 102 upregulated genes (75 genes in BPCa and 27 genes in LPCa). (C) A scatter plot of 75 genes upregulated in BPCa cells co‐cultured with MG63 cells evaluated by sample 4 (BPCa cells co‐culture with MG63 cells) vs. sample 3 (BPCa cell mono‐culture) and sample 4 (BPCa cells co‐culture with MG63 cells) vs. sample 2 (LPCa cells co‐culture with MG63 cells) from Fig. S3B. *n* = 3. (D) Top 8 upregulated genes selected in BPCa cells by horizontal co‐culture with MG63 cells. The expression of all eight genes in PCa cells (DU145, 22RV‐1, C4‐2, and C4‐2B) with/without MG63 co‐culture (OB) and with/without RAW246.7 co‐culture (OC) is shown as TPM by RNA‐seq. *n* = 3. All error bars indicate SD.

In a bone metastasis environment, both cancer cells and bone resident cells are mutually stimulated. To investigate key factors responsible for osteoblastic bone metastasis, we searched for genes (factor X) that were specifically upregulated in BPCa cells in the interaction with OB‐like cells, while this interaction did not occur between LPCa cells and OB‐like cells (Fig. S3A). For this purpose, we set up the co‐culture system and performed RNA sequencing in BPCa cells alone, LPCa cells alone, BPCa cells with OB‐like cells, and LPCa cells with OB‐like cells. Briefly, DU145 (LPCa), 22Rv1 (LPCa), C4‐2 (BPCa), and C4‐2B (BPCa) cells were co‐cultured with MG63 cells in a horizontal co‐culture system, NICO‐1, for 5 days, and total RNA was extracted from PCa cell lysates for RNA sequencing (Fig. S3B). PCA of the whole transcriptome showed three clusters that were divided into “DU145,” “22Rv1,” and “C4‐2, C4‐2B”, except for one sample of DU145 co‐cultured with MG63 cells (Fig. S4A). In Venn diagrams (Fig. 2A), we sought to identify genes whose expression was changed only in co‐culture with BPCa cells and MG63 cells. In co‐culture with MG63 cells, 75 genes (twofold and *P* < 0.05) were specifically expressed in BPCa cells, and 27 genes (twofold and *P* < 0.05) were specifically expressed in LPCa cells (Fig. 2A,B). Of the 75 genes upregulated in BPCa cells, SERPINA3 and LCN2 were remarkably expressed in BPCa cells co‐cultured with MG63 cells (Fig. 2C). The eight most highly expressed genes are shown (Fig. 2D), and BPCa cells (both C4‐2 and C4‐2B) co‐cultured with MG63 cells clearly overexpressed SERPINA3 and LCN2. On the other hand, from 27 genes upregulated in LPCa cells, MSANTD3‐TMEFF1 and GAL3ST1 were found to be upregulated (Fig. S4B). In this study, because only BPCa cells positively influenced OB‐like cells, we focused on SERPINA3 and LCN2 expression in BPCa cells.

### 3.4 Validation of specific SERPINA3 and LCN2 expression in BPCa cells with OB‐like cells and human bone metastatic PCa specimens

**Fig. 3:**
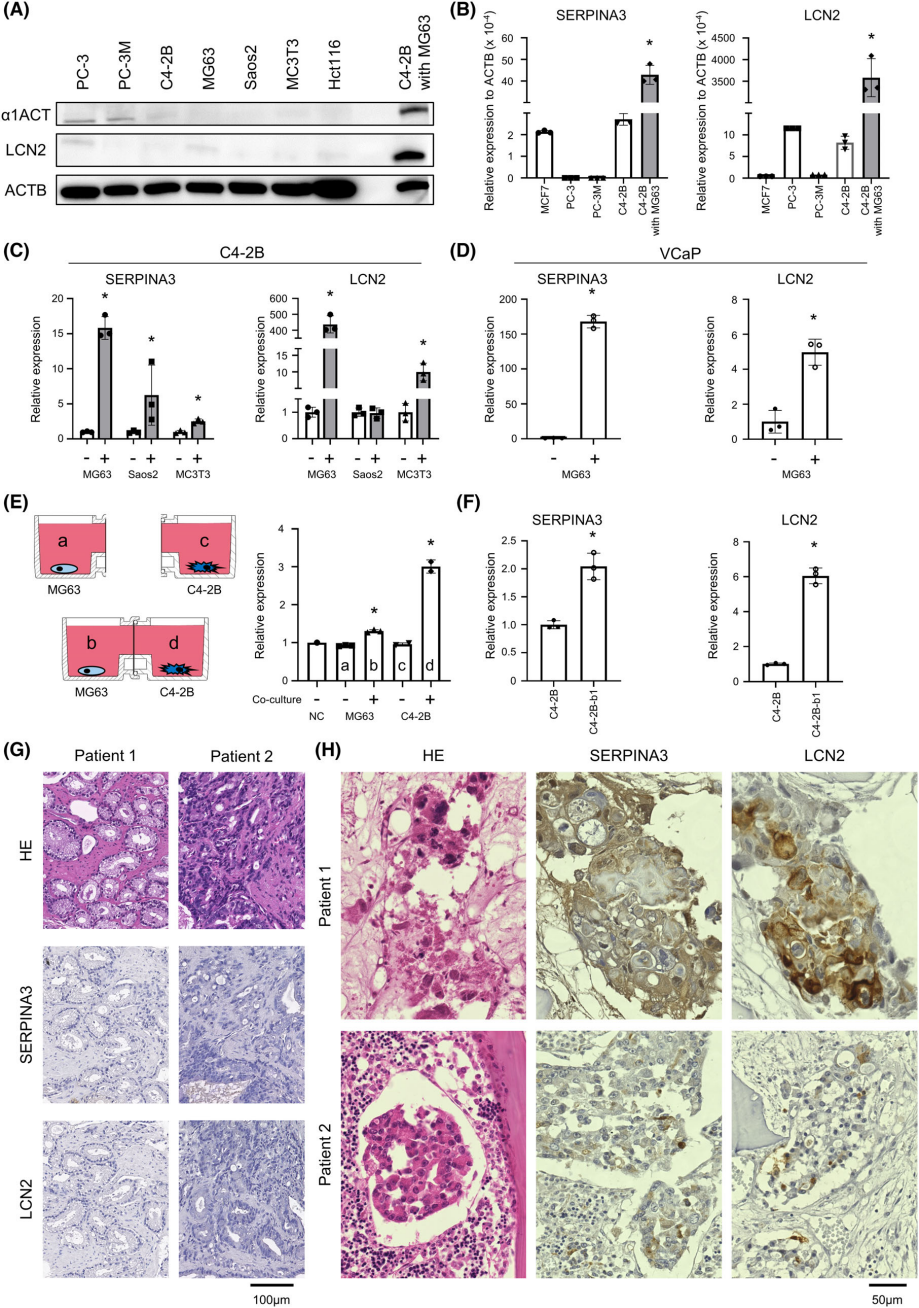
Validation of SERPINA3 and LCN2 expression and secretion in BPCa cells. (A) Protein expression levels of α1ACT, alpha‐1‐antichymotrypsin (SERPINA3), and LCN2 in PC‐3, PC‐3M, C4‐2B, MG63, Saos2, MC3T3, Hct116, and C4‐2B co‐cultured with MG63 cells measured by immunoblotting. *n* = 3. (B) RNA expression levels of SERPINA3 and LCN2 in MCF7, PC‐3, PC‐3M, and C4‐2B cells measured by qRT–PCR. *n* = 3, *P < 0.05; based on analysis of variance (ANOVA). (C) RNA expression levels of SERPINA3 and LCN2 in C4‐2B cells with and without OBs (MG63, Saos2, and MC3T3) in horizontal co‐culture. *n* = 3, *P < 0.05; based on Student's *t* test. (D) RNA expression levels of SERPINA3 and LCN2 in another BPCa cell line, VCaP, with and without MG63 cells in horizontal co‐culture. *n* = 3, *P < 0.05; based on Student's *t* test. (E) Schematic protocol and protein expression levels of α1ACT in the CM from horizontal co‐culture of C4‐2B and MG63 cells measured by ELISAs. a: CM collected from MG63 cells mono‐culture well, b: CM collected from MG63 cells co‐culture with C4‐2B cells well, c: CM collected from C4‐2B cells mono‐culture well, d: CM collected from C4‐2B cells co‐culture with MG63 cells well. *n* = 3, *P < 0.05; based on analysis of variance (ANOVA). (F) RNA expression levels of SERPINA3 and LCN2 in C4‐2B and C4‐2B‐b1 cells that were isolated from a xenograft tumor of bone metastasis. *n* = 3, *P < 0.05; based on Student's *t* test. (G) Representative microscopic images of HE (left), SERPINA3 (middle), and LCN2 (right) staining of human PCa tumors from primary sites. The experiment was repeated three times. Scale bar indicates 100 μm. (H) Representative microscopic images of HE (left), SERPINA3 (middle), and LCN2 (right) staining of human PCa tumors from bone metastasis sites. All error bars indicate SD. The experiment was repeated three times. Scale bar indicates 50 μm.

The protein and RNA expression levels of SERPINA3 (known as α1ACT, alpha‐1‐antichymotrypsin) and LCN2 were specifically detected in C4‐2B co‐cultured with MG63 cells (Fig. 3A,B), although almost no expression of these proteins and RNAs in other mono‐cultured cells was found (Fig. 3A,B). To validate the expression of SERPINA3 and LCN2 in other models, we co‐cultured C4‐2B cells with other OB‐like cells, such as Saos2 and MC3T3 cells, in a Boyden chamber. Consistent with the MG63 results, co‐culture of C4‐2B cells with either Saos2 or MC3T‐3 cells clearly induced SERPINA3 and LCN2 expression (Fig. 3C and Fig. S5A). We also confirmed the overexpression of SERPINA3 and LCN2 in co‐culture of MG63 and VCaP, another BPCa cell line (Fig. 3D and Fig. S5B). As α1ACT is a secreted protein, we examined α1ACT levels in the CM (medium “d”) in co‐culture system. As expected, C4‐2B cells with MG63 cells showed a high level of α1ACT (Fig. 3E). A slightly high level of α1ACT was also detected in the CM (medium “b”) from the MG63 cells treated with C4‐2B cells (Fig. 3E). These results suggested that co‐culturing with OBs *in vitro* markedly increased SERPINA3 and LCN2 expression in BPCa cells.

We also investigated the overexpression of SERPINA3 and LCN2 in a bone metastatic animal model (Fig. S6). For the *in vivo* experiment, we established a bone metastatic subline of C4‐2B using caudal artery injection, which selectively causes bone metastasis. The established bone metastatic C4‐2B‐b1 cells showed higher expression of SERPINA3 and LCN2 than the parental C4‐2B cells (Fig. 3F). This result suggested that BPCa cells were induced to promote SERPINA3 and LCN2 in mouse bone metastasis environment.

To validate these results in human specimens, we performed IHC with antibodies against SERPINA3 and LCN2. These proteins were not stained in primary PCa (Fig. 3G and Fig. S7A), while they were positive in metastatic PCa cells at bone (Fig. 3H and Fig. S7B). These data suggested that BPCa cells were specifically stimulated to express SERPINA3 and LCN2 by OBs in human bone metastasis environment.

### 3.5 Interaction mechanism between BPCa cells and OBs via OB EVs

**Fig. 4:**
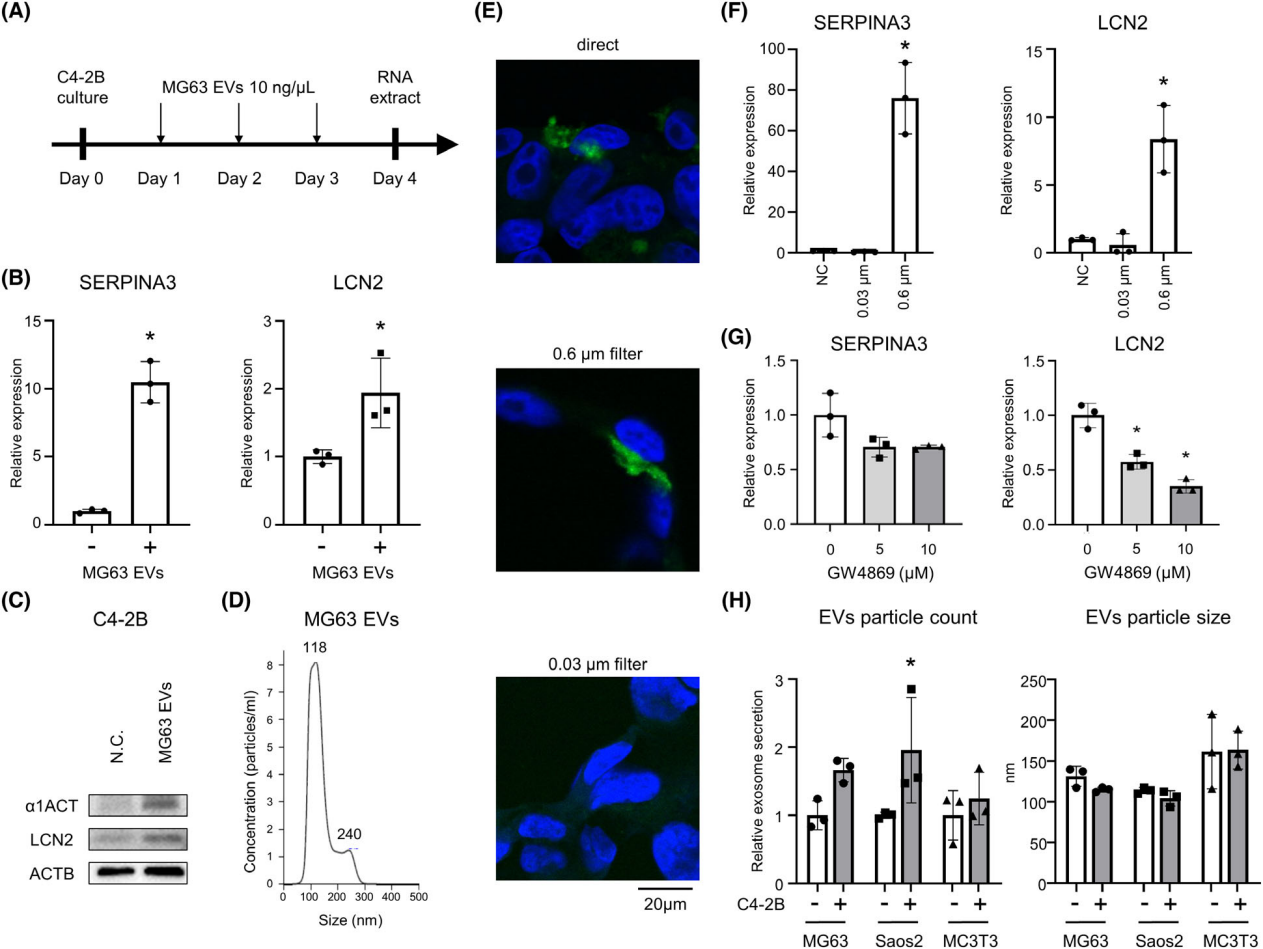
Intercellular communications between BPCa cells and OBs via OB EVs. (A) Schematic protocol examining SERPINA3 and LCN2 levels in C4‐2B cells with and without MG63 EVs. (B) RNA expression levels of SERPINA3 and LCN2 in C4‐2B cells with and without MG63 EVs. *n* = 3, *P < 0.05; based on Student's *t* test. (C) Protein expression levels of α1ACT (alpha‐1‐antichymotrypsin: SERPINA3 transcription factor) and LCN2 in C4‐2B cells with and without MG63 EVs measured by immunoblotting. *n* = 3. (D) Particle numbers and sizes of MG63 EVs analyzed by NanoSight. The experiment was repeated three times. (E) Representative confocal microscopic images. MG63 EVs were labeled with PKH67 green and added to the horizontal co‐culture wells. C4‐2B cell nuclei were stained with DAPI. A schematic protocol is shown in Fig. S8. The experiment was repeated three times. Scale bar indicates 20 μm. (F) RNA expression of SERPINA3 and LCN2 in C4‐2B cells using a 0.03 or 0.6 μm filter in the horizontal co‐culture system. *n* = 3, *P < 0.05; based on analysis of variance (ANOVA). (G) RNA expression of SERPINA3 and LCN2 in C4‐2B cells vertically co‐cultured with MG63 cells with/without GW4869 (5 or 10 μm). *n* = 3, *P < 0.05; based on analysis of variance (ANOVA). (H) EV secretion amount and particle size were measured with ExoScreen and NanoSight, respectively. Osteoblast‐like cells, such as MG63, Saos2, and MC3T3, were cultured in Boyden lower wells with C4‐2B cells in the upper wells for 5 days co‐culture. After 5‐day co‐culture, OB‐like cells were passaged to 12‐well plate and cultured for 4 days (advanced DMEM was used for the last 2 days). The CM was collected for ExoScreen and NanoSight analysis. *n* = 3, *P < 0.05; based on Student's *t* test. All error bars indicate SD.

Our data suggested that OBs stimulate BPCa cells via OB humoral factors; however, its molecular basis has not been determined. Recently, EVs have been shown to be an important intercellular communication tool among cells [22]. In this study, we investigated whether OB EVs are key factors that induce SERPINA3 and LCN2 in BPCa cells. To evaluate the effect of OB EVs, we collected EVs from MG63‐CM by ultracentrifugation. C4‐2B cells were treated with 10 ng·μL^−1^ MG63 EVs every day and cultured for 4 days (Fig. 4A). SERPINA3 and LCN2 expression was significantly upregulated by MG63 EVs (Fig. 4B,C). To evaluate whether OB EVs were involved in SERPINA3 and LCN2 expression in BPCa cells, we cultured C4‐2B cells in the horizontal co‐culture system NICO‐1 with a 0.6 or 0.03 μm filter (Fig. S8A–C). EVs are known to be 50–200 nm in size [23], and MG63 EVs are approximately 118 nm in size (Fig. 4D), so they can pass through a 0.6 μm filter but theoretically cannot pass through a 0.03 μm filter. PKH67‐labeled MG63 EVs were added as shown in Fig. S8A–C. MG63 EVs were incorporated into C4‐2B cells by direct addition (Fig. 4E top and Fig. S8A) and indirect addition with a 0.6 μm filter (Fig. 4E middle and Fig. S8B). No EVs were detected in C4‐2B cells when they were indirectly added with a 0.03 μm filter (Fig. 4E bottom and Fig. S8C). Next, we examined the effect of MG63 EVs in co‐culture of MG63 and C4‐2B cells (Fig. 4F and Fig. S8D,E). After 5 days of co‐culture, RNA was extracted from C4‐2B cells, and SERPINA3 and LCN2 levels were measured by qRT–PCR. As expected, SERPINA3 and LCN2 expression was significantly upregulated in co‐culture with a 0.6 μm filter compared with mono‐cultures and co‐cultures with a 0.03 μm filter. These results clearly indicate that MG63 EVs specifically stimulated SERPINA3 and LCN2 expression in BPCa cells. To further assess the effect of EVs, GW4869, an EV secretion inhibitor was used [24]. Suppression of MG63 EV with GW4869 slightly reduced expression of SERPINA3 and significantly reduced expression of LCN2 (Fig. 4G). Additionally, we found co‐culturing with BPCa cells enhanced EV secretion in OB‐like cells, such as MG63, Saos2, and MC3T3 cells (Fig. 4H left); however, the EV sizes were not significantly changed (Fig. 4H right). From these results, BPCa cells stimulated OBs to secrete EVs that induced SERPINA3 and LCN2 in BPCa cells, suggesting a positive feedback loop between BPCa cells and OBs.

### 3.6 Functional analysis of SERPINA3 and LCN2 in OBs and OCs

To evaluate the effect of SERPINA3 and LCN2 on OBs, we generated an overexpression model in C4‐2B cells (Fig. S9A right). Overexpressed C4‐2B cells were seeded on Boyden upper wells, and OB‐like cells were seeded on lower wells for 5 days of co‐culture. No significant changes in MG63 and Saos2 proliferation were observed after SERPINA3 or LCN2 overexpression in BPCa cells, while MC3T3 was slightly affected by both (Fig. S9B). Next, we evaluated OB differentiation by detecting OB differentiation markers with qRT–PCR. LCN2 significantly increased Alpl, and other OB differentiation markers were slightly increased. Slight increase in early OB differentiation markers was observed in the SERPINA3‐overexpressing cells (Fig. S9C).

To evaluate the effect of SERPINA3 and LCN2 on OC proliferation, we collected the CM of the SERPINA3‐ or LCN2‐overexpressing HEK293T cells and administered it to RAW264.7 cells with/without sRANKL (50 ng·mL^−1^) (Fig. S10A). No notable changes in RAW264.7 cells were observed in either the SERPINA3‐ or LCN2‐CM with/without sRANKL (Fig. S10B). Next, we examined OC differentiation by counting TRAP‐positive multinucleated OC numbers. SERPINA3‐CM decreased the differentiated OC numbers (Fig. S10C). We also confirmed OC differentiation by qRT–PCR using the Boyden chamber system (upper well: SERPINA3‐ or LCN2‐overexpressing C4‐2B cells and lower well: RAW264.7 cells) with sRANKL (50 ng·mL^−1^). In this experiment, most OC differentiation markers in RAW264.7 cells decreased in the co‐culturing of both the SERPINA3‐ and LCN2‐overexpressing C4‐2B cells (Fig. S10D). Collectively, these data suggested that SERPINA3 and LCN2 in BPCa cells might suppress OC differentiation, presumably leading to indirect osteogenesis.

### 3.7 *In vivo* analysis of SERPINA3 and LCN2 in bone metastasis

**Fig. 5:**
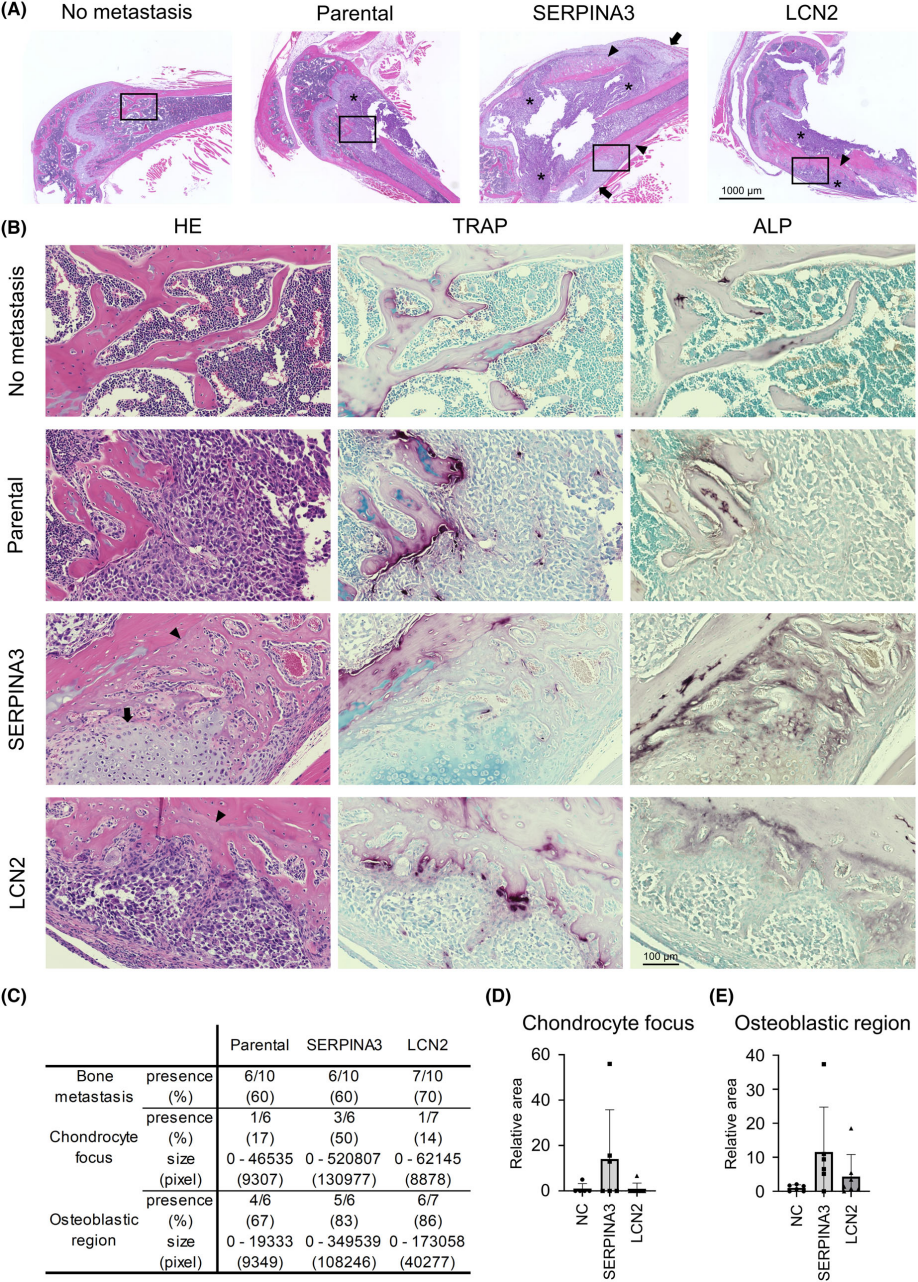
SERPINA3 and LCN2 overexpression causes osteogenesis in bone metastasis of PCa. (A) Representative images of H&E‐stained paraffin sections of normal bone and bone metastatic sites in xenografted mice (SERPINA3 and LCN2). * Indicates PCa cells. Arrows indicate the chondrocyte foci. Arrowheads indicate the osteoblastic region. The experiment was repeated two times. Scale bar indicates 1000 μm. (B) Representative images of H&E‐, TRAP‐, and ALP‐stained paraffin sections magnified from A section (marked by rectangle). The experiment was repeated two times. Scale bar indicates 100 μm. (C) A table showing demographics of bone metastatic sites in the xenografted mice classified by cell type (parental, SERPINA3, and LCN2). (D) Relative area counts of chondrocyte foci in bone metastatic sites of xenografted mice (WT vs. SERPINA3; *P* = 0.1832). *n* = 10, *P < 0.05; based on analysis of variance (ANOVA). (E) Relative area counts of osteoblastic regions in bone metastatic sites of xenografted mice (WT vs. SERPINA3; *P* = 0.0796). *n* = 10, *P < 0.05; based on analysis of variance (ANOVA). All error bars indicate SD.

We examined the roles of SERPINA3 and LCN2 *in vivo*. To investigate the effects of SERPINA3 and LCN2 on bone metastasis *in vivo*, we established SERPINA3‐ or LCN2‐overexpressing PC‐3M cells (Fig. S9 left). PC‐3M cells are LPCa cells, and we aimed to evaluate whether overexpression of SERPINA3 or LCN2 converted the osteolytic to osteoblastic phenotype in the cells. SERPINA3‐ or LCN2‐overexpressing PC‐3M cells were injected via the caudal artery and generated femoral or tibial bone metastasis. Bone metastasis was monitored until 4 weeks after injection by IVIS based on luminescence, as luciferase genes were lentivirally introduced in PC‐3M cells (Fig. S11A). Mice were sacrificed at 4 weeks, and then, the femur and tibia were sectioned for H&E stain and IHC for ALP, TRAP, SERPINEA3, and LCN2. The overexpression of SERPINA3 and LCN2 was confirmed with IHC (Fig. S11B). In the bone metastasis of the parental PC‐3M cells, there were no obvious osteoblastic areas, and part of the cortical and trabecular bone was lost with OCs beside the bone matrix, indicating osteolytic bone metastasis (Fig. 5A,B). Conversely, bone metastases from the SERPINA3‐ and LCN2‐overexpressing PC‐3M cells showed some osteoblastic regions adjacent to the bone matrix (Fig. 5A,B). In these osteoblastic regions, the bone matrix and OBs were stained with ALP (brown regions). No such findings were observed in normal mouse bone trabeculae with no metastatic cancer cells, suggesting that these osteoblastic regions were not preexisting at bone trabeculae but were areas of new bone formation. Specifically, in bone metastases from the SERPINA3‐overexpressing PC‐3M cells, TRAP staining showed very few OCs (purple‐stained cells) at the site of osteogenesis (Fig. 5B), consistent with *the in vitro* results showing that SERPINA3 inhibited OC differentiation. Additionally, the SERPINA3‐overexpressing PC‐3M cells generated chondrocyte foci adjacent to the newly formed osteoblastic regions. The area of the chondrocyte foci and osteoblast regions were calculated for each slide of bone metastasis (Fig. 5C). Chondrocyte foci were observed in 50% of the SERPINA3‐overexpressing bone metastases; in contrast, they were rare and small in the parental and LCN2‐overexpressing bone metastases (Fig. 5C,D). Osteoblastic regions were found in most bone metastases, but these regions were larger in the LCN2‐ and especially SERPINA3‐overexpressing bone metastases (Fig. 5C,E). These results indicated that both SERPINA3 and LCN2 might contribute to the osteoblastic phenotype.

### 3.8 Effects of SERPINA3 and LCN2 on PCa cells

**Fig. 6:**
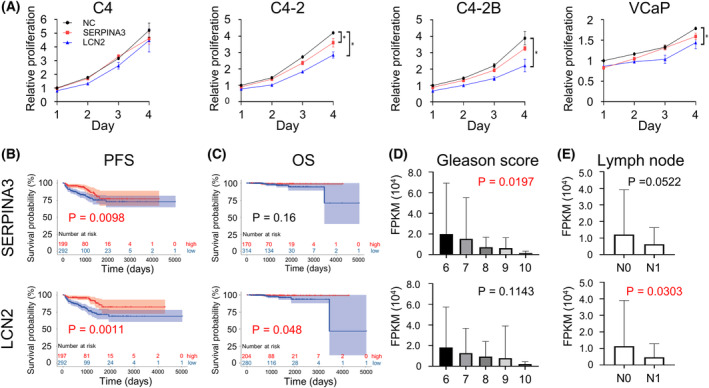
SERPINA3 and LCN2 effects on BPCa cell proliferation and cohort characteristic analysis of SERPINA3 and LCN2 in PCa patients. (A) Relative proliferation rates of BPCa cells (C4, C4‐2, C4‐2B, and VCaP) treated with CM from SERPINA3‐ or LCN2‐overexpressing HEK293T cells shown in Fig. S10A. *n* = 4, *P < 0.05; based on analysis of variance (ANOVA). (B, C) Kaplan–Meier plots with SERPINA3 and LCN2 expression profiles from TCGA PCa dataset using PFS (progression‐free survival: PSA failure) status (B) and OS status (C). Low expression of SERPINA3 correlated with poor prognosis of PFS (high, *n* = 199; low, *n* = 292; *P* = 0.0098). Low LCN2 expression correlated with poor prognosis of PFS (high, *n* = 197; low, *n* = 294; *P* = 0.00011) and OS (high, *n* = 204; low, *n* = 280; *P* = 0.048). Based on log‐rank test. (D, E) RNA expression levels of SERPINA3 and LCN2 in TCGA PCa dataset based on Gleason score (D; based on analysis of variance (ANOVA)) and lymph node metastasis (E; based on Student's *t* test). All error bars indicate SD. FPKM, fragments per kilobase of exon per million reads mapped; PSA, prostate‐specific antigen.

Thus far, we investigated the effects of SERPINA3 and LCN2 on the bone metastasis phenotype. Finally, we assessed the roles of SERPINA3 and LCN2 in PCa itself. To evaluate the effects of SERPINA3 and LCN2 on PCa, we collected CM from the SERPINA3‐ or LCN2‐overexpressing HEK293T cells and administered the medium to four types of BPCa cells, C4, C4‐2, C4‐2B, and VCaP (Fig. S10A). BPCa cell proliferation was markedly suppressed by LCN2 and slightly suppressed by SERPINA3 (Fig. 6A and Fig. S12A). No differences in caspase‐3 and ‐7 activity were observed with SERPINA3 and LCN2 expression (Fig. S12B). Both SERPINA3 and LCN2 overexpression in C4‐2B cell also significantly suppressed proliferation (Fig. S12C). Invasion assay, wound healing assay, and colony formation assay was performed for SERPINA3 or LCN2 or both overexpressed C4‐2B cells (Fig. S12D–F). Invading cells were reduced by SERPINA3, and colony formation was promoted by LCN2. These results indicated that SERPINA3 and LCN2, which are highly expressed in BPCa cells via OB interactions, suppress BPCa cell growth in an autocrine manner.

We next evaluated the clinical relevance of SERPINA3 and LCN2 expression in PCa. TCGA data analysis of 550 PCa patients [25] revealed that high expression of SERPINA3 or LCN2 was significantly correlated with better PFS (Fig. 6B), while only LNC2 was correlated with better OS (Fig. 6C). In the high Gleason score group (generally an aggressive phenotype), both SERPINA3 and LCN2 expression levels were low (Fig. 6D). The group with lymph node metastasis at initial diagnosis had low expression of both SERPINA3 and LCN2 (Fig. 6E). Overall, these results suggested that SERPINA3 and LCN2 might act as tumor suppressors in PCa.

Finally, we examined the feasibility of SERPINA3 (α1ACT) and LCN2 as biomarkers. We collected 35 plasma samples from PCa patients at the National Cancer Center Hospital, Japan (Table S2), and the protein levels of α1ACT and LCN2 were measured by ELISA (Fig. S13A). The protein level of α1ACT was significantly higher in both the osteoblastic bone metastasis and osteolytic bone metastasis groups than in the local group (Fig. S13A upper panel). LCN2 was significantly higher in the osteolytic bone metastasis group than in the local and osteoblastic bone metastasis groups (Fig. S13A lower panel). No significant change was found in PFS or OS based on SERPINA3 and LCN2 plasma concentrations (Fig. S13B,C). These results suggested that α1ACT and LCN2 might be biomarkers for bone metastasis of PCa but could not distinguish osteoblastic bone metastasis from others.

## 4 Discussion

In this study, we revealed several important phenomena in BPCa bone metastasis. BPCa cells stimulated OBs to secrete EVs, which in turn specifically stimulated BPCa cells to express extremely high levels of SERPINA3 and LCN2. SERPINA3 and LCN2 promoted osteoblastic bone metastasis by suppressing OCs, differentiating Obs, and creating chondrocyte foci. Additionally, SERPINA3 and LCN2 suppress BPCa cell proliferation in an autocrine manner, suggesting that they may act as tumor suppressors in bone metastatic PCa. These results may explain the enigmatic formation of the osteoblastic phenotype in bone metastases and the favorable prognosis of BPCa. Thus, SERPINA3 and LCN2 may serve as prognostic markers and therapeutic targets.

We demonstrated an interaction between PCa cells and osteogenic cells such as OBs and OCs in a horizontal co‐culture system. RNA sequencing (RNA‐seq) analysis showed unique SERPINA3 and LCN2 upregulation in BPCa cells by co‐culture with OBs, which was not found using a vertical co‐culture system. The interaction between cancer cells and bone resident cells has been evaluated by the administration of humoral factors or vertical co‐culture experiments in recent reports. PSA, IGF1, PDGFA, MDA‐BF‐1, PDGFB, FGF1, ET‐1, BMP2, BMP4, FGF2, uPA, and TGFβ are known as osteoblastic factors secreted from PCa cells [19]. These factors were not clearly upregulated in BPCa cells compared to LPCa cells in our results; thus, they may be involved in bone formation but are not major factors in the osteoblastic bone metastasis phenotype. The vertical co‐culture system only allows us to investigate a one‐directional effect between cancer cells and bone resident cells. For specific elevation of SERPINA3 and LCN2, the two‐directional interaction between BPCa cells and OBs was essential. Our horizontal co‐culture system better represents bone metastasis in PCa than the conventional vertical co‐culture system.

We found that SERPINA3 and LCN2 mainly influenced OCs and OBs, respectively; SERPINA3 inhibited OC differentiation, and LCN2 promoted OB differentiation. SERPINA3 was reported as a serine protease inhibitor targeting pancreatic chymotrypsin, leukocyte cathepsin G, mast cell chymase, human glandular kallikrein 2, kallikrein 3 (also known as prostate‐specific antigen), pancreatic cationic elastase, and lung serum protease [26]. Although the specific molecular mechanism is still unknown, we directly showed the effect of SERPINA3 on the inhibition of OC differentiation. One report by Akbar et al. showed inhibition of OC formation via α1‐antitrypsin, a transcript of SERPINA1 [27]; however, no reports have shown a relationship between SERPINA3 and OC formation thus far. On the other hand, LCN2 is known to be widely expressed in neutrophils, adipocytes, and bone marrow. This molecule belongs to the lipocalin superfamily and is involved in many functions, such as inflammation, chronic renal failure, energy metabolism, and tumor association, but the role of LCN2 in bone metabolism is still unclear [28, 29]. In this study, we showed that LCN2 promoted OB differentiation. Several studies supported our findings and revealed that LCN2 was involved in OB differentiation from bone‐marrow‐derived MSCs [30, 31]. In addition, Capulli et al. reported reduced ALP‐positive colonies in bone‐marrow‐derived MSCs from LCN2 knockout mice, as well as reduced OB and bone volume in LCN2 knockout mice [32], although the same research group reported controversial results [33]. Collectively, LCN2 plays a positive role in OB differentiation, leading to bone formation.

SERPINA3 stimulated the bone environment, and chondrocyte foci and new osteoblastic regions were found next to each other. This phenomenon suggests a key role in creating osteoblastic bone metastasis. To our knowledge, this is the first study to demonstrate chondrocyte foci in a bone metastasis environment. Endochondral ossification and intramembranous ossification are well‐known bone formations that occur during bone development, and bone remodeling occurs in parallel with ossification [34]. In endochondral ossification, which primarily occurs at tubular bone, enlarged chondrocytes degenerate or die, and osteogenesis occurs by newly arrived OBs along with angiogenesis. In intramembranous ossification, which primarily occurs at flat bone, osteogenic cells differentiate directly into OBs, independent of chondrocyte differentiation, resulting in osteogenesis. Besides, Yasui et al. reported transchondroid bone formation, which differs from the two major ossifications [35, 36]. Moriwaki et al. reported transchondroid bone formation in bone metastasis from PCa and breast cancer patients [37, 38]. From our results of SERPINA3‐overexpressing PCa bone metastasis, chondrocyte foci appeared adjacent to newly created osteoblastic regions, in which OBs and blood vessels were included. Thus, SERPINA3‐overexpressing PCa bone metastasis is not typical transchondroid bone formation but rather closely resembles endochondral ossification. Our data may imply that chondrocyte induction is an important factor in the formation of osteoblastic bone metastases, suggesting that ossification is a more critical event than bone remodeling. This finding could explain why co‐culture of OBs with SERPINA3‐overexpressing PCa cells did not result in significant changes in OB differentiation. Instead of an interaction between OBs and PCa cells, an interaction between bone‐marrow‐derived MSCs and PCa cells would be required to examine chondrocyte differentiation. Many studies have focused on the interaction between cancer cells and OBs or OCs to explore the bone metastasis phenotype. However, these studies cannot evaluate the possibility of endochondral ossification in creating a bone metastasis phenotype. Toyoda et al. and Boeuf et al. reported higher expression of SERPINA3 in chondrocytes than in MSCs [39, 40]. Although the molecular mechanism remains unclear, SERPINA3 is suggested to be an important factor in chondrocyte differentiation leading to osteoblastic bone metastasis in PCa. Unlike SERPINA3, LCN2 showed a new osteoblastic region, but no chondrocyte foci were found. These results suggest that OB differentiation by LCN2 causes osteoblastic bone metastasis, which is distinct from the SERPINA3 mechanism.

Our *in vitro* experiments showed that SERPINA3 and LCN2 suppressed the proliferation of BPCa cells, suggesting that these two genes are tumor suppressors in BPCa; TCGA dataset analysis supported this conclusion. In fact, both SERPINA3 and LCN2 were reported as both oncogenes and tumor suppressors in cancer, and their roles are likely to be tissue specific [41, 42]. For example, Xing et al. [43] reported that SERPINA3 inhibits apoptosis of LPCa cells (PC‐3 and DU145), and Tung et al. [44] reported that knockdown of LCN2 suppressed the growth and invasion of LPCa cells (PC‐3 and DU145), while we evaluated the function of these genes using BPCa cells. In contrast, Jiang et al. [45] reported that mast cell chymase stimulates lung cancer and decreases E‐cadherin, leading to tumor cell detachment, cell migration, and apoptosis. Laface et al. [46] reported that mast cell chymase stimulates angiogenesis in pancreatic cancer. Thus, as SERPINA3 is known to inhibit mast cell chymase [26], SERPINA3 may act as a tumor suppressor. Likewise, Lee et al. reported that LCN2 suppressed the JNK and PI3K/Akt pathways to inhibit the proliferation of human hepatocellular carcinoma cells [47], and Kim et al. also reported that LCN2 suppressed the RAS/ERK pathway to inhibit the proliferation of breast cancer cells [48]. Because the PI3K/Akt or RAS/ERK pathway is activated in BPCa cells, such as the C4 sublines and VCaP [49], it is speculated that LCN2 blocks this pathway and inhibits growth. Analysis of the TCGA dataset, which includes data from 550 PCa patients, revealed that PTEN, a suppressor of the PI3K/Akt pathway, is the most deleted or mutated gene at 17% [25]. Most human PCas are BPCa, and activation of the PI3K/Akt pathway via PTEN loss is presumably a major characteristic of BPCa. As LCN2 is known to suppress the PI3K/Akt pathway, LCN2 could be a tumor suppressor in BPCa.

Because both SERPINA3 and LCN2 are secreted proteins, it is also important to consider the feasibility of plasma biomarkers. Plasma analysis from NCC, Japan, suggested α1ACT as a biomarker of bone metastasis in PCa and LCN2 as a biomarker of osteolytic bone metastasis in PCa. This result did not clearly reflect our data showing that α1ACT and LCN2 were specifically increased in BPCa. NCC patient plasma analysis has some limitations: (a) the cohort size in the analysis was small, (b) some of the osteolytic group patients had both osteolytic and osteoblastic bone metastasis, and (c) α1ACT and LCN2 are known as biomarkers of other diseases. Plasma α1ACT is known as a biomarker of cardiovascular disease, tuberculosis, Alzheimer's disease, dementia, ovarian cancer, and pancreatic cancer [42]. Plasma LCN2 is known as a biomarker of acute kidney injury, arthritis, acute pancreatitis, obesity, cardiovascular disease, and multiple sclerosis [28]. These diseases were excluded from the analysis, but unknown effects from other diseases are inevitable.

Several cytokines and receptor proteins included inside or on the membrane of OB‐derived EVs were suggested as a mediator for stimulating BPCa to express SERPINA3 and LCN2. Cytokines (TGFβ, TNFα, IL‐1, 6, and OSM) are known as a mediator of SERPINA3 by activating NF‐κB signaling, MAPK/Erk signaling, and Jak/Stat signaling [50]. Cytokines (TNFα, IL‐1,6,17, and IFNγ) and growth factors such as EGF are known as a mediator of LCN2 by activating NF‐κB signaling, MAPK/Erk signaling, and Jak/Stat signaling [51, 52, 53]. MG63 cells highly expressed both ligands and receptors of TGFβ, TNFα, IL‐1, 6, 17, OSM, TNFα, IFNγ, and EGF (Fig. S14A). All these pathways were upregulated in C4‐2B cells by co‐culturing with MG63 cells (Fig. S14B). These results indicate that cytokines (TGFβ, IL, IFN, TNF, and OCM) and growth factors such as EGF contained within MG63 EVs or membrane proteins such as cytokines and growth factor receptors on MG63 EVs were delivered to C4‐2B cells to express SERPINA3 and LCN2.

## 5 Conclusions

**Fig. 7:**
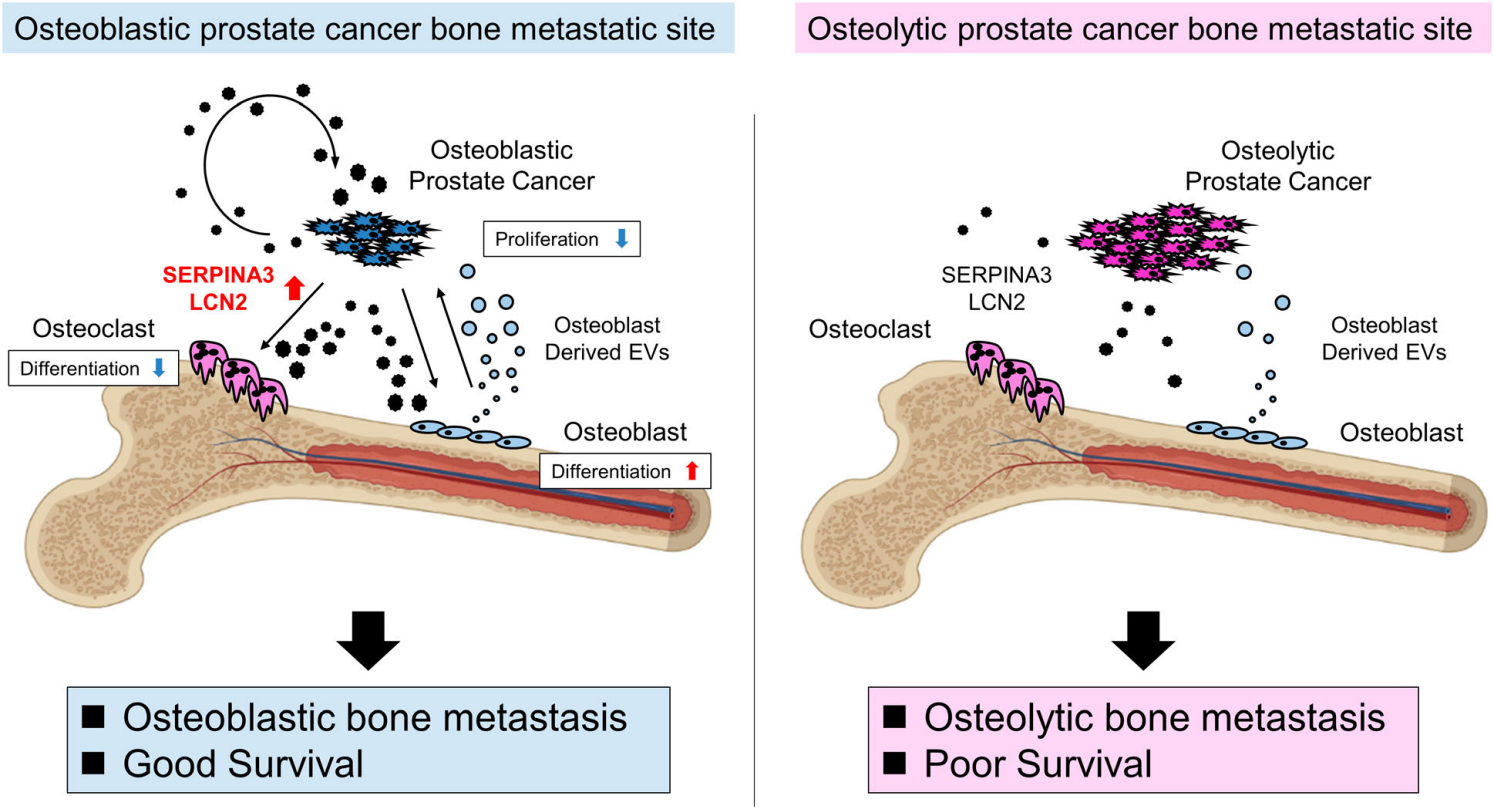
Schema of the bone metastasis environment phenotype distinguished by SERPINA3 and LCN2 functions in PCa, OCs, and OBs. SERPINA3 and LCN2 are upregulated in BPCa via osteoblast‐derived EVs. These factors create osteoblastic bone metastases by stimulating osteoblasts and osteoclasts to act as osteogenesis. In addition, these factors suppress PCa cell growth, leading to favorable survival. In LPCa, this mechanism is absent, resulting in osteolytic bone metastases and poor survival.

We showed SERPINA3 and LCN2 upregulation in BPCa via OB‐derived EVs in PCa bone metastasis environment. We revealed that SERPINA3 and LCN2 contributed to creating osteoblastic bone metastasis and suppressing PCa cell growth, leading to good survival (Fig. 7). In LPCa, this mechanism is absent, resulting in osteolytic bone metastases and poor survival. Thus, our findings suggest that SERPINA3 and LCN2 could be diagnostic biomarkers for predicting bone phenotype and survival.

## Conflict of interest

The authors declare no conflict of interest.

## Author contributions

KI, TO, and YY conceived the idea of the study. KI, TY, and YH performed the experiments. SS analyzed and interpreted the data regarding pathology, created figures, and wrote the text regarding pathology. KI and JN developed the statistical analysis plan and conducted statistical analyses. FU and TS helped to design experiments. EN, YM, HF, TK, and SE collected human samples for experiments. KI drafted the original manuscript. All authors reviewed the manuscript draft and revised it critically on intellectual content. All authors approved the final version of the manuscript to be published.

### Peer review

The peer review history for this article is available at https://www.webofscience.com/api/gateway/wos/peer‐review/10.1002/1878‐0261.13484.

## Supporting information


**Fig. S1.** NCC patient data analysis between bone metastasis phenotypes. (A) Overall survival in patients with local PCa, BPCa, and LPCa. (B) Plots for age, Gleason score, PSA, NSE and ALP in patients with local PCa, BPCa, and LPCa. *P<0.05.

**Fig. S2.** OC responses by horizontal co‐culture with LPCa or BPCa cells. (A) Proliferation rates of RAW246.7 cells with and without PCa cells (LPCa or BPCa) in horizontal co‐culture. (B) Expression of OC differentiation marker genes in RAW246.7 cells with and without PCa cells (LPCa or BPCa) measured by qRT–PCR. n = 3, *P<0.05. c Representative microscopic images of RAW246.7 cells with 50 ng/mL sRANKL in horizontal co‐culture with and without PCa cells (LPCa or BPCa). The arrow indicates differentiated OCs. n = 3.

**Fig. S3.** Schema and concept for detecting factor X by the horizontal co‐culture of PCa and MG63 cells. (A) Schema of the bone metastasis environment. The function of the assumed factor X in PCa, OC, and OB cells is shown in both osteoblastic and osteolytic prostate cancer bone metastasis environments. (B) Schematic protocol of horizontal co‐culture to collect RNA samples for RNA‐seq analysis.

**Fig. S4.** PCA and upregulated genes in LPCa cells co‐cultured with MG63 cells. (A) PCA plot of the whole transcriptome from the samples in Supplementary Figure 3B. (B) Scatter plot of 27 genes upregulated in LPCa (DU145 and 22Rv1) cells co‐cultured with MG63 cells evaluated by sample‐2 (LPCa cells co‐culture with MG63 cells) vs. sample‐1 (LPCa cell mono‐culture) and sample‐2 (LPCa cells co‐cultured with MG63 cells) sample‐4 (BPCa (C4‐2 and C4‐2B) cells co‐culture with MG63 cells).

**Fig. S5.** α1ACT and LCN2 protein expression in BPCa cells. (A) Protein expression levels of α1ACT and LCN2 in C4‐2B cells with and without OBs (MG63 and Saos2) in horizontal co‐culture cells measured by immunoblotting. n = 3. (B) Protein expression levels of α1ACT and LCN2 in VCaP cells with and without MG63 in horizontal co‐culture cells measured by immunoblotting. n = 3.

**Fig. S6.** Establishment of bone metastatic PCa from C4‐2B cells via caudal artery injection. Schematic protocol for creating bone metastasis in an osteoblastic prostate cancer‐derived xenograft mouse model. Tumor cells were isolated from bone metastasis lesions and named C4‐2B‐luc‐b1.

**Fig. S7.** Microscopic images of SERPINA3 and LCN2 in human prostate cancer (patient 3–5). (A) Representative microscopic images of HE (left), SERPINA3 (middle), and LCN2 (right) staining of human prostate cancer tumors from primary sites. (B) Representative microscopic images of HE (left), SERPINA3 (middle), and LCN2 (right) staining of human prostate cancer tumors from bone metastasis sites.

**Fig. S8.** Schematic protocol for vertical co‐culture. (A, B, and C) Schematic protocol for detecting MG63 EVs in C4‐2B cells through a filter of 0.03 μm or 0.6 μm. Confocal microscopic images are shown in Figure 4D. (A) Direct EV treatment, (B) co‐culture with a 0.6 μm filter, (C) co‐culture with a 0.03 μm filter. (D and E) Schematic protocol and RNA expression of SERPINA3 and LCN2 in C4‐2B cells using a 0.03 μm or 0.6 μm filter in the horizonal co‐culture system. n = 3, *P<0.05.

**Fig. S9.** SERPINA3 and LCN2 effects on OB‐like cells. (A) Protein levels of α1ACT and LCN2 with and without SERPINA3 and LCN2 overexpression in PC‐3 M and C4‐2B cells by immunoblotting. n = 3. (B) OB‐like cell (MG63, Saos2, and MC3T3) proliferation in vertical co‐culture with SERPINA3‐ and LCN2‐overexpressing C4‐2B cells by luminescent cell viability assays. n = 3, *P<0.05. (C) RNA expression levels of OB differentiation marker genes (Col1a1, Alpl, Ibsp, Spp1, and Bglap) in MC3T3 cells vertically co‐cultured with SERPINA3‐ and LCN2‐overexpressing C4‐2B cells by qRT–PCR. NC: negative control. n = 3, *P<0.05.

**Fig. S10.** SERPINA3 and LCN2 effects on RAW246.7 cells. (A) Schematic protocol of the luminescent cell viability assays of BPCa cells (C4, C4‐2, C4‐2B, and VCaP) with and without SERPINA3‐ and LCN2‐conditioned medium from HEK293T cells. (B) Proliferation rates of RAW246.7 cells. SRANKL (0 ng/mL or 50 ng/mL) was added on Day 0. n = 3. (C) Cell number counts of OCs. Multinuclear cells containing three or more TRAP‐positive nuclei were counted as OCs. n = 3, *P<0.05. (D) RNA expression levels of OC differentiation marker genes (Trap, Ctsk, Dc‐stamp, Rank, Mitf, Nfatc1, and Prdm1) in RAW246.7 cells. RAW246.7 cells were vertically co‐cultured with SERPINA3‐ and LCN2‐overexpressing C4‐2B cells by qRT–PCR. n = 3, *P<0.05. NC: negative control.

**Fig. S11.** Xenografted PCa cells at bone metastasis. (A) Representative *in vivo* bioluminescence imaging of xenografted mice 4 weeks after caudal artery injection of PC‐3M cell lines (PC‐3M, PC‐3M‐SERPINA3 and PC‐3M‐LCN2). (B) Representative images of SERPINA3‐ and LCN2‐stained paraffin sections in bone metastatic sites.

**Fig. S12.** SERPINA3 and LCN2 effects on BPCa. (A) Luminescent cell viability assays of BPCa cells (C4, C4‐2, C4‐2B, and VCaP) with 10 and 25 μl/well of SERPINA3‐ or LCN2‐conditioned medium from HEK293T cells. n = 4, *P<0.05. (B) The relative caspase 3/7 activity in BPCa cells (C4, C4‐2, C4‐2B, and VCaP) treated with SERPINA3‐ or LCN2‐conditioned medium from HEK293T cells. Caspase 3/7 activity was measured after 24 hours of treatment. n = 4, *P<0.05. (C) C4‐2B cell lines (C4‐2B, C4‐2B‐SERPINA3, C4‐2B‐LCN2, and C4‐2B‐SERPINA3‐LCN2) proliferation measured by luminescent cell viability assays. n = 12, *P<0.05. (D) Invasion assay of C4‐2B cell line cultured in collagen‐coated Boyden chambers. n = 6, *P<0.05. (E) Wound healing assay of C4‐2B cell lines. n = 6, *P<0.05. (F) Soft agar colony formation assay of C4‐2B cell lines. n = 3, *P<0.05.

**Fig. S13.** α1 ACT and LCN2 plasma concentration in PCa patients. (A) α1ACT and LCN2 concentrations in PCa patients without bone metastasis, with osteoblastic bone metastasis (OB) and with osteolytic bone metastasis (OL) from NCC patient plasma examined by an ELISA kit. *P<0.05 (B and C) Kaplan–Meier plots with SERPINA3 and LCN2 protein levels from NCC prostate cancer patient plasma using PFS (B) and overall survival (C). No significant correlation was found between plasma SERPINA3 or LCN2 concentrations and PFS or OS.

**Fig. S14.** Upstream mediator candidates of SERPINA3 and LCN2. (A) TPM counts of MG63 cells RNA expression from RNA‐seq data in GEO database (GSE208277). (B) Significantly upregulated pathways in C4‐2B cells by horizontal co‐culturing with MG63 cells (C4‐2B co‐cultured with MG63 vs. C4‐2B). Ingenuity Pathway Analysis (IPA, https://www.digital‐biology.co.jp/allianced/products/ipa/) was performed to identify upstream regulators.

**Table S1.** Primer sequences for qRT‐PCR.

**Table S2.** Baseline demographics of patients from the National Cancer Center, Japan.

## Data Availability

The data generated in this study are available in Gene Expression Omnibus (GEO) at GSE210729.
