# Supplementary figures and images for: Osteoblast‐derived extracellular vesicles exert osteoblastic and tumor‐suppressive functions via SERPINA3 and LCN2 in prostate cancer
Source: Mol Oncol. 2023 Aug 4;17(10):2147–67. doi: 10.1002/1878-0261.13484 (PMC10552899; doi:10.1002/1878-0261.13484)

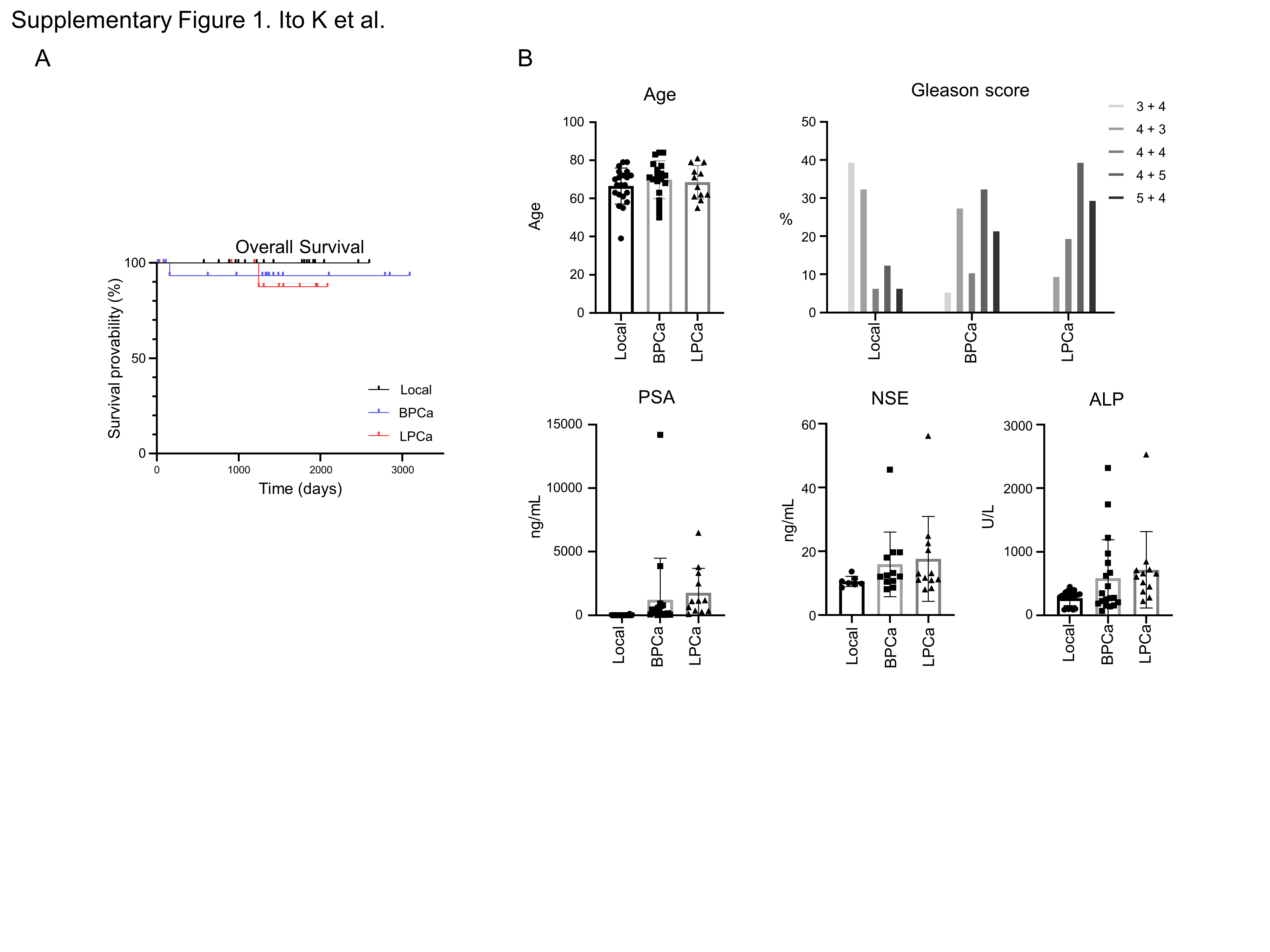

Supplement: Supplementary file 1 — Fig. S1. NCC patient data analysis between bone metastasis phenotypes. (A) Overall survival in patients with local PCa, BPCa, and LPCa. (B) Plots for age, Gleason score, PSA, NSE and ALP in patients with local PCa, BPCa, and LPCa. *P<0.05. [file MOL2-17-2147-s007.tif]

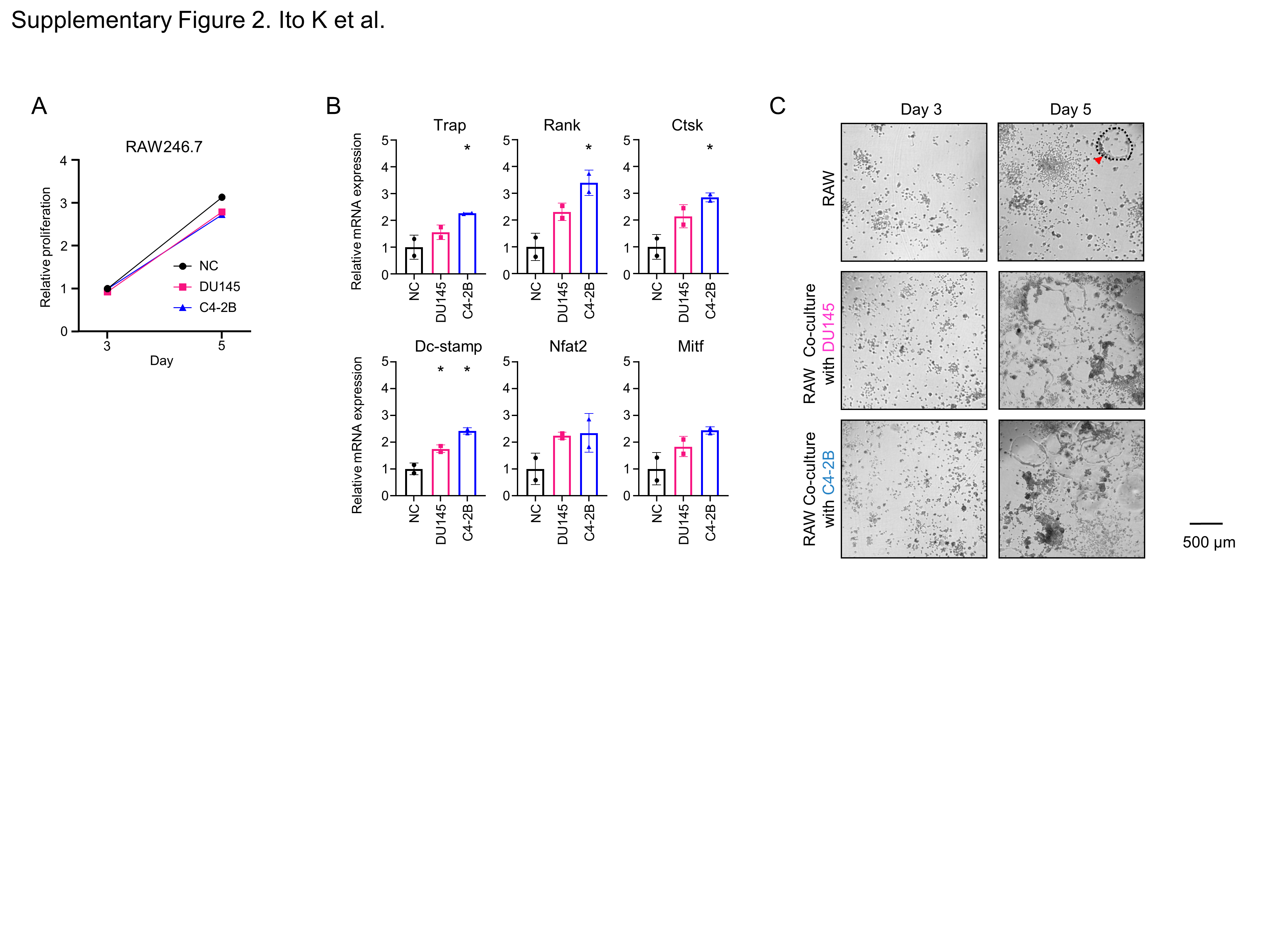

Supplement: Supplementary file 2 — Fig. S2. OC responses by horizontal co‐culture with LPCa or BPCa cells. (A) Proliferation rates of RAW246.7 cells with and without PCa cells (LPCa or BPCa) in horizontal co‐culture. (B) Expression of OC differentiation marker genes in RAW246.7 cells with and without PCa cells (LPCa or BPCa) measured by qRT–PCR. n = 3, *P<0.05. c Representative microscopic images of RAW246.7 cells with 50 ng/mL sRANKL in horizontal co‐culture with and without PCa cells (LPCa or BPCa). The arrow indicates differentiated OCs. n = 3. [file MOL2-17-2147-s002.tif]

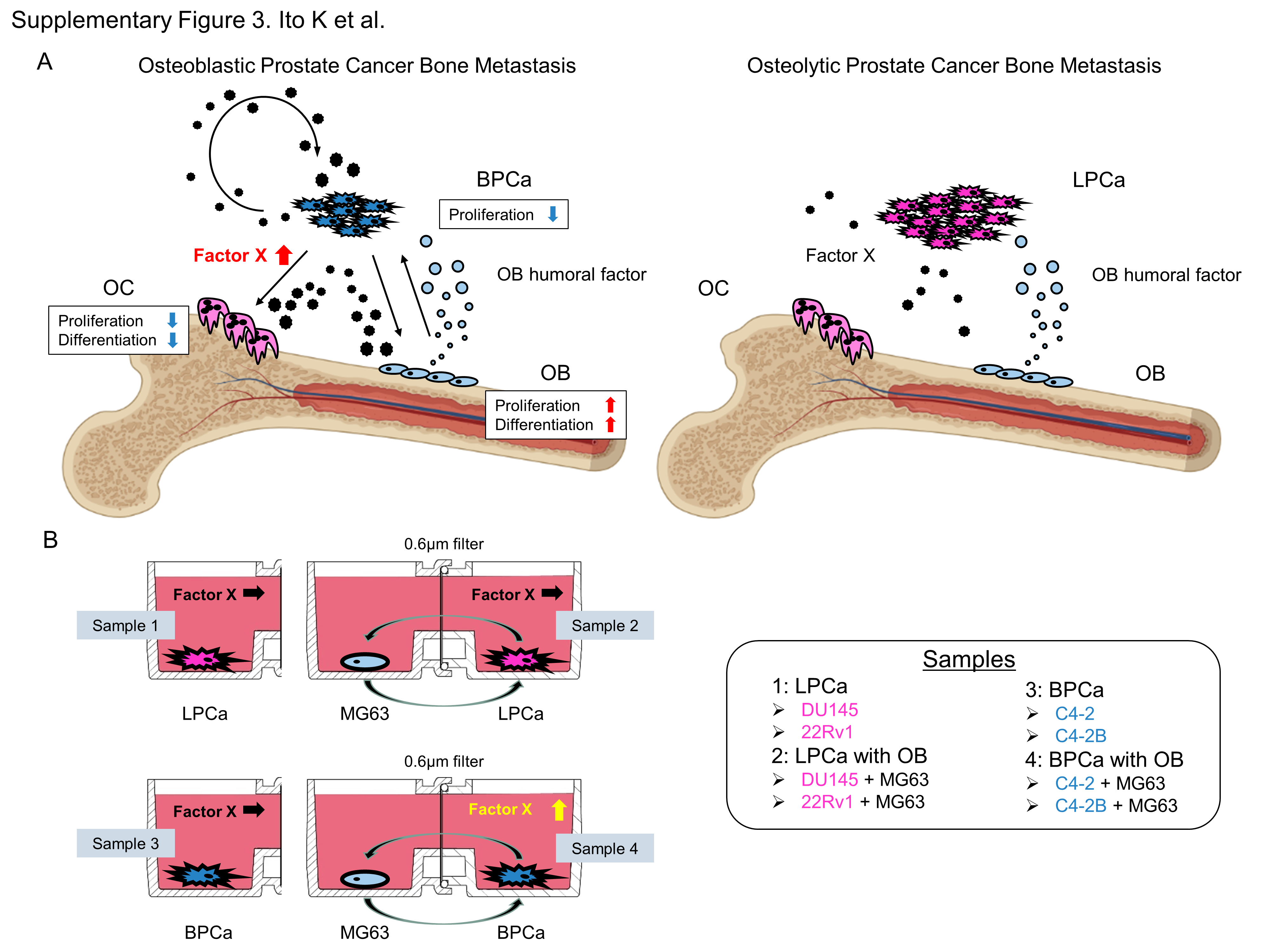

Supplement: Supplementary file 3 — Fig. S3. Schema and concept for detecting factor X by the horizontal co‐culture of PCa and MG63 cells. (A) Schema of the bone metastasis environment. The function of the assumed factor X in PCa, OC, and OB cells is shown in both osteoblastic and osteolytic prostate cancer bone metastasis environments. (B) Schematic protocol of horizontal co‐culture to collect RNA samples for RNA‐seq analysis. [file MOL2-17-2147-s012.tif]

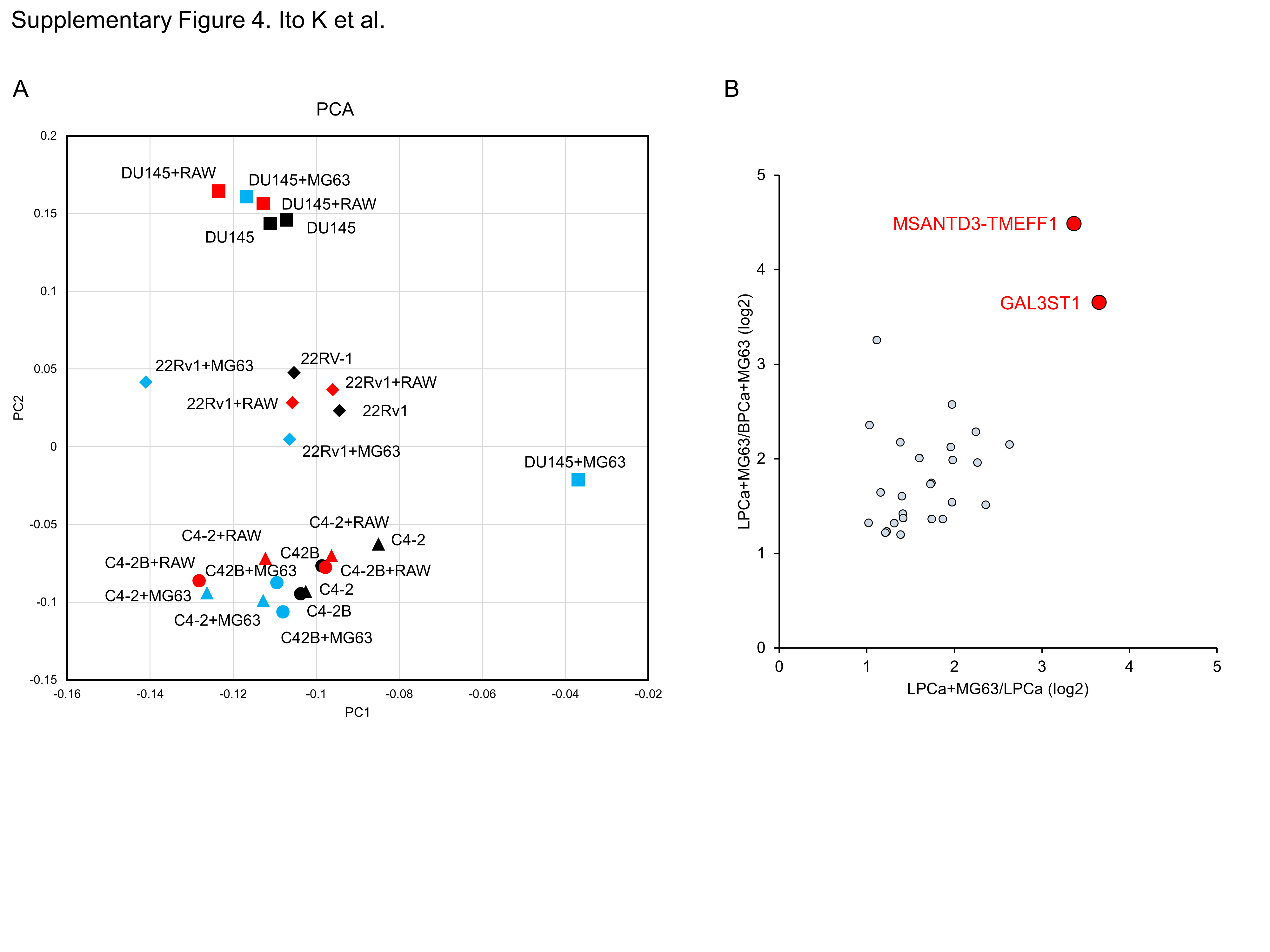

Supplement: Supplementary file 4 — Fig. S4. PCA and upregulated genes in LPCa cells co‐cultured with MG63 cells. (A) PCA plot of the whole transcriptome from the samples in Supplementary Figure 3B. (B) Scatter plot of 27 genes upregulated in LPCa (DU145 and 22Rv1) cells co‐cultured with MG63 cells evaluated by sample‐2 (LPCa cells co‐culture with MG63 cells) vs. sample‐1 (LPCa cell mono‐culture) and sample‐2 (LPCa cells co‐cultured with MG63 cells) sample‐4 (BPCa (C4‐2 and C4‐2B) cells co‐culture with MG63 cells). [file MOL2-17-2147-s011.tif]

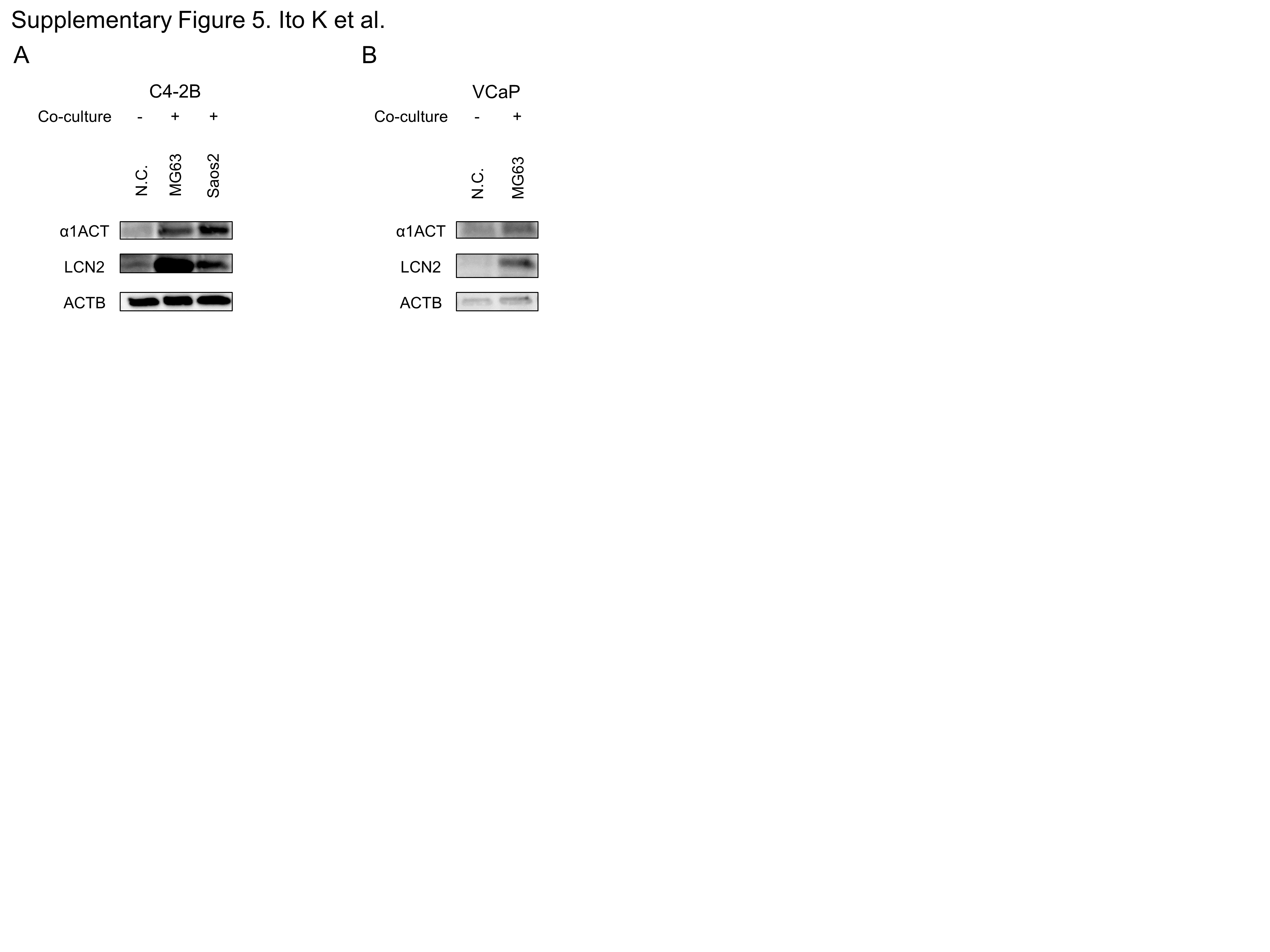

Supplement: Supplementary file 5 — Fig. S5. α1ACT and LCN2 protein expression in BPCa cells. (A) Protein expression levels of α1ACT and LCN2 in C4‐2B cells with and without OBs (MG63 and Saos2) in horizontal co‐culture cells measured by immunoblotting. n = 3. (B) Protein expression levels of α1ACT and LCN2 in VCaP cells with and without MG63 in horizontal co‐culture cells measured by immunoblotting. n = 3. [file MOL2-17-2147-s016.tif]

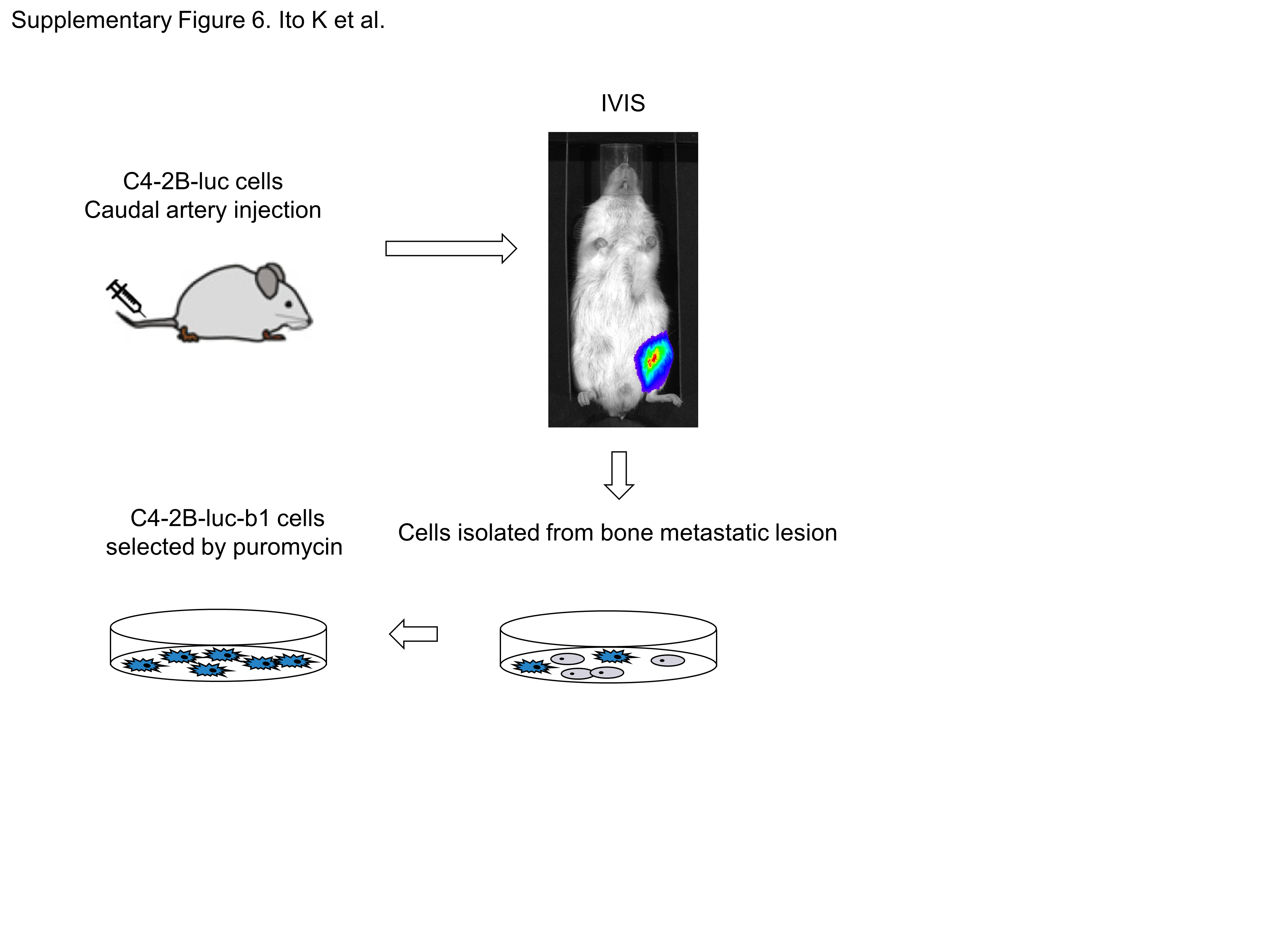

Supplement: Supplementary file 6 — Fig. S6. Establishment of bone metastatic PCa from C4‐2B cells via caudal artery injection. Schematic protocol for creating bone metastasis in an osteoblastic prostate cancer‐derived xenograft mouse model. Tumor cells were isolated from bone metastasis lesions and named C4‐2B‐luc‐b1. [file MOL2-17-2147-s014.tif]

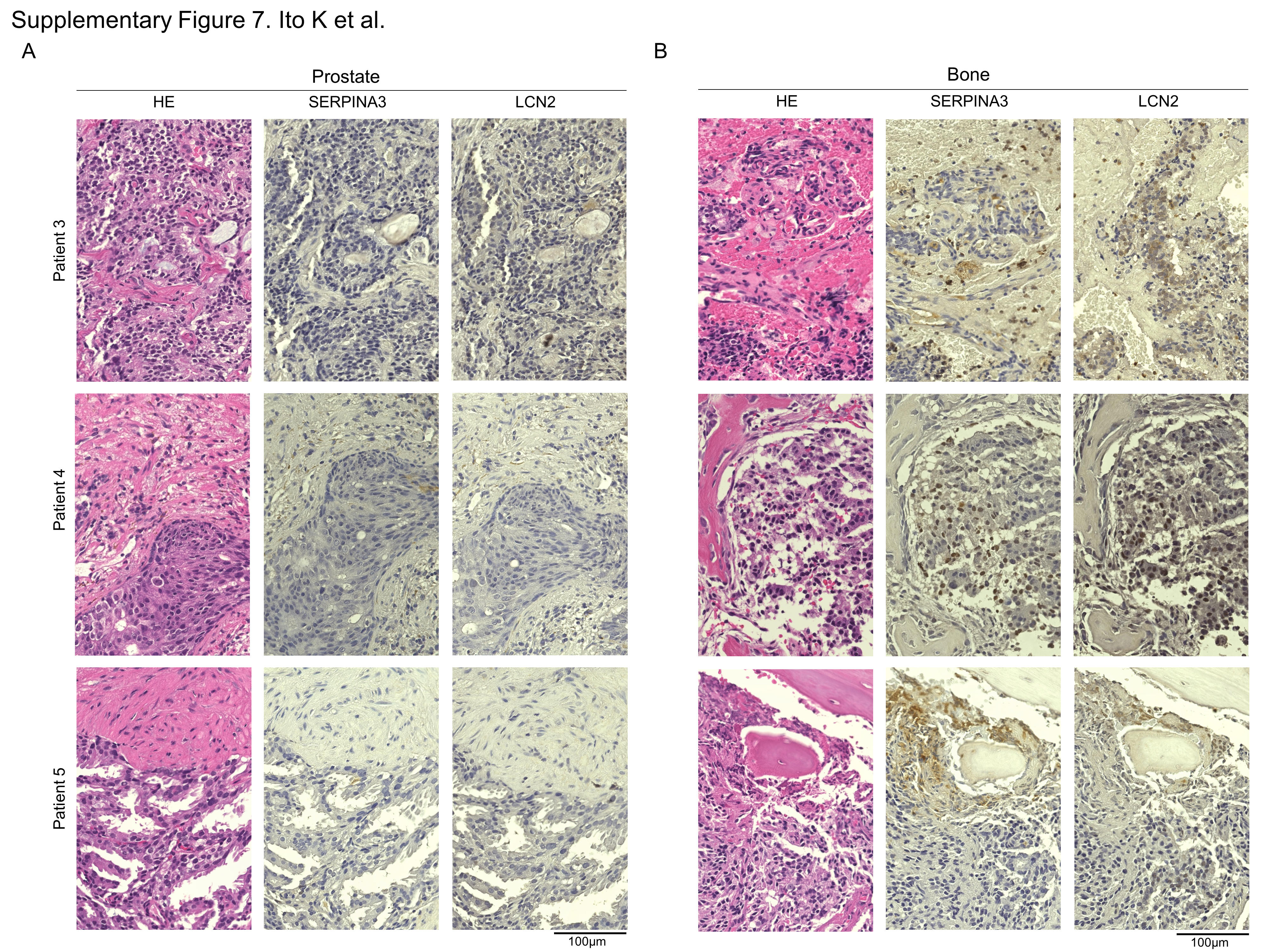

Supplement: Supplementary file 7 — Fig. S7. Microscopic images of SERPINA3 and LCN2 in human prostate cancer (patient 3–5). (A) Representative microscopic images of HE (left), SERPINA3 (middle), and LCN2 (right) staining of human prostate cancer tumors from primary sites. (B) Representative microscopic images of HE (left), SERPINA3 (middle), and LCN2 (right) staining of human prostate cancer tumors from bone metastasis sites. [file MOL2-17-2147-s008.tif]

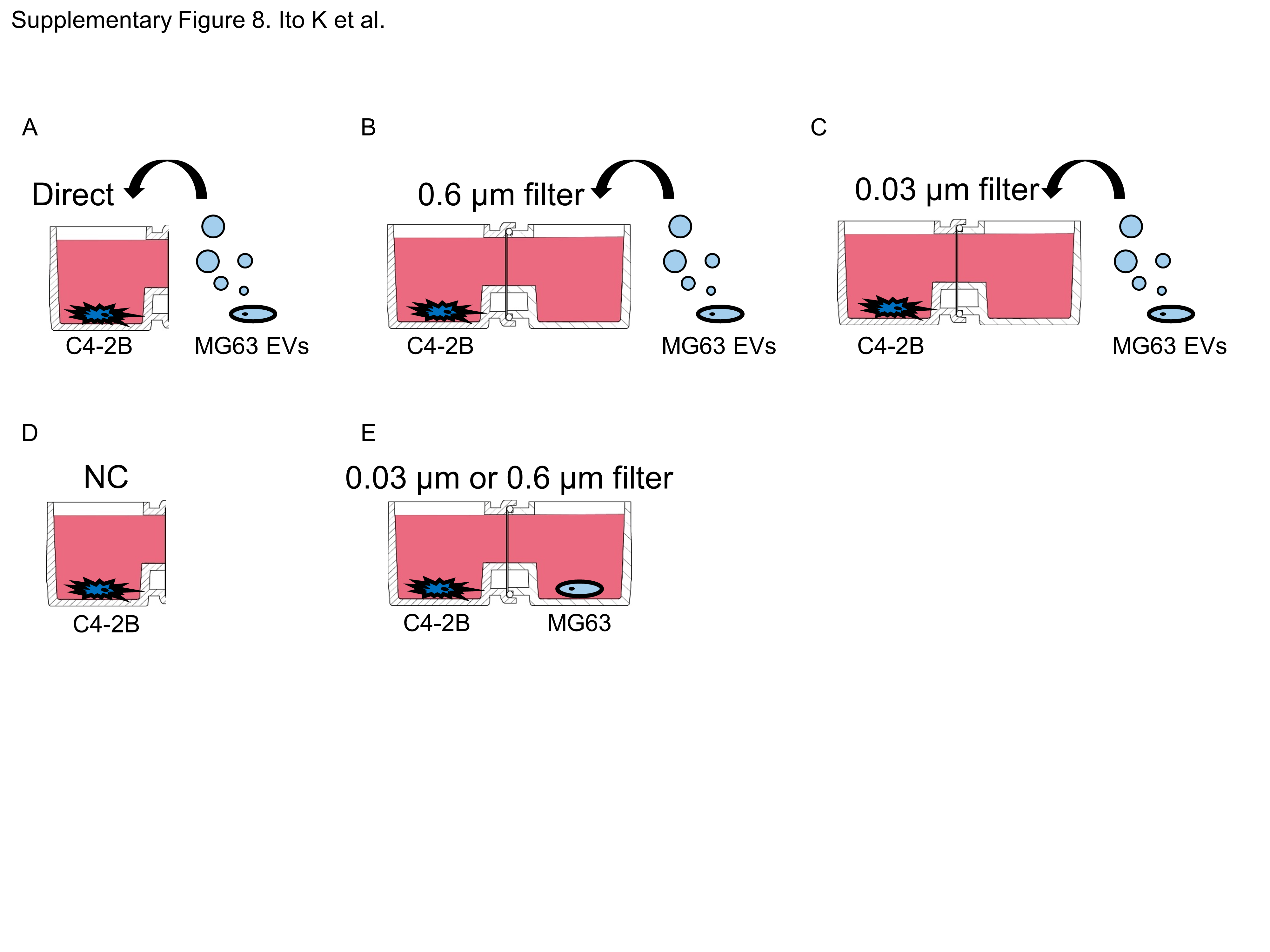

Supplement: Supplementary file 8 — Fig. S8. Schematic protocol for vertical co‐culture. (A, B, and C) Schematic protocol for detecting MG63 EVs in C4‐2B cells through a filter of 0.03 μm or 0.6 μm. Confocal microscopic images are shown in Figure 4D. (A) Direct EV treatment, (B) co‐culture with a 0.6 μm filter, (C) co‐culture with a 0.03 μm filter. (D and E) Schematic protocol and RNA expression of SERPINA3 and LCN2 in C4‐2B cells using a 0.03 μm or 0.6 μm filter in the horizonal co‐culture system. n = 3, *P<0.05. [file MOL2-17-2147-s004.tif]

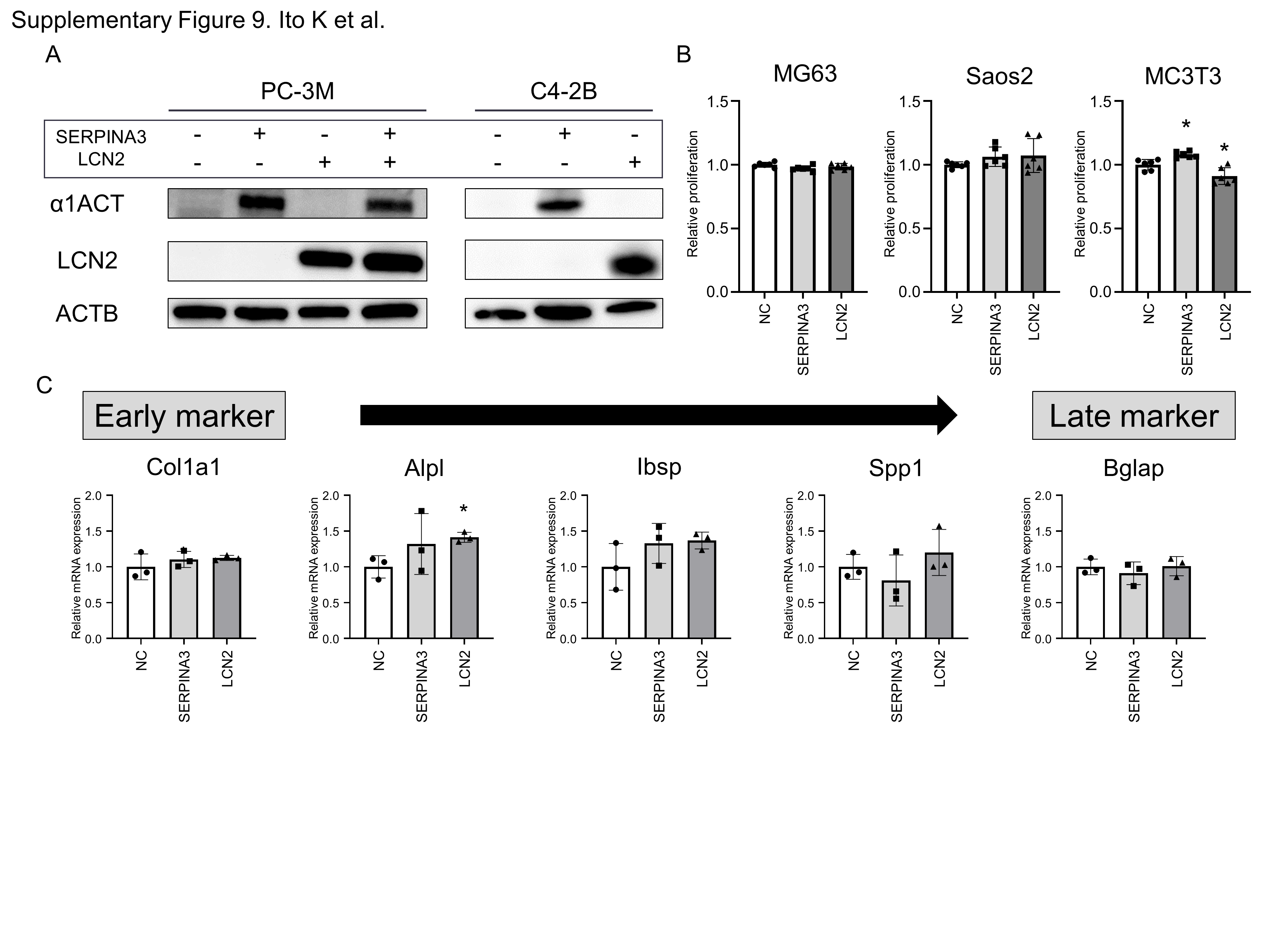

Supplement: Supplementary file 9 — Fig. S9. SERPINA3 and LCN2 effects on OB‐like cells. (A) Protein levels of α1ACT and LCN2 with and without SERPINA3 and LCN2 overexpression in PC‐3 M and C4‐2B cells by immunoblotting. n = 3. (B) OB‐like cell (MG63, Saos2, and MC3T3) proliferation in vertical co‐culture with SERPINA3‐ and LCN2‐overexpressing C4‐2B cells by luminescent cell viability assays. n = 3, *P<0.05. (C) RNA expression levels of OB differentiation marker genes (Col1a1, Alpl, Ibsp, Spp1, and Bglap) in MC3T3 cells vertically co‐cultured with SERPINA3‐ and LCN2‐overexpressing C4‐2B cells by qRT–PCR. NC: negative control. n = 3, *P<0.05. [file MOL2-17-2147-s009.tif]

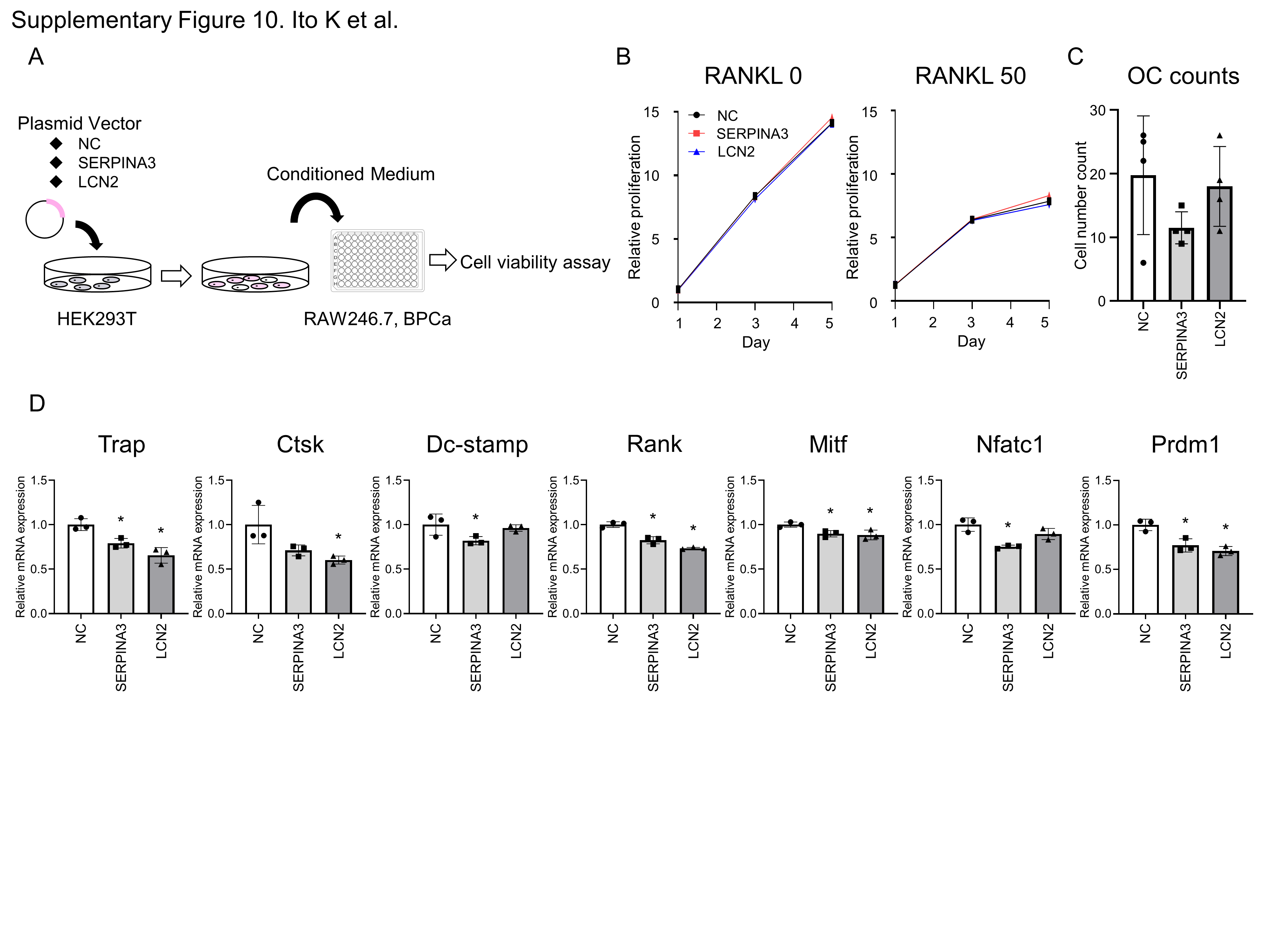

Supplement: Supplementary file 10 — Fig. S10. SERPINA3 and LCN2 effects on RAW246.7 cells. (A) Schematic protocol of the luminescent cell viability assays of BPCa cells (C4, C4‐2, C4‐2B, and VCaP) with and without SERPINA3‐ and LCN2‐conditioned medium from HEK293T cells. (B) Proliferation rates of RAW246.7 cells. SRANKL (0 ng/mL or 50 ng/mL) was added on Day 0. n = 3. (C) Cell number counts of OCs. Multinuclear cells containing three or more TRAP‐positive nuclei were counted as OCs. n = 3, *P<0.05. (D) RNA expression levels of OC differentiation marker genes (Trap, Ctsk, Dc‐stamp, Rank, Mitf, Nfatc1, and Prdm1) in RAW246.7 cells. RAW246.7 cells were vertically co‐cultured with SERPINA3‐ and LCN2‐overexpressing C4‐2B cells by qRT–PCR. n = 3, *P<0.05. NC: negative control. [file MOL2-17-2147-s005.tif]

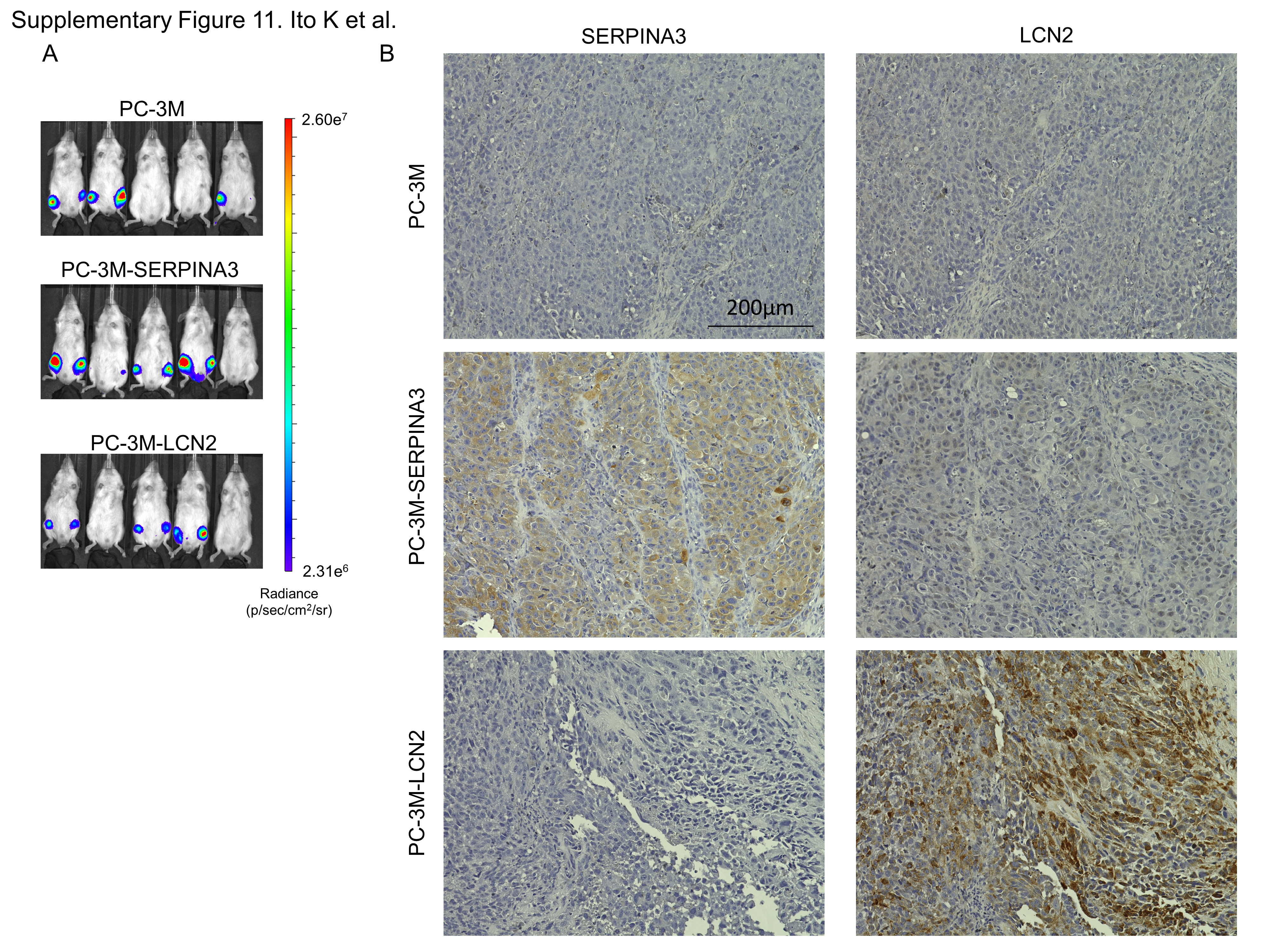

Supplement: Supplementary file 11 — Fig. S11. Xenografted PCa cells at bone metastasis. (A) Representative in vivo bioluminescence imaging of xenografted mice 4 weeks after caudal artery injection of PC‐3M cell lines (PC‐3M, PC‐3M‐SERPINA3 and PC‐3M‐LCN2). (B) Representative images of SERPINA3‐ and LCN2‐stained paraffin sections in bone metastatic sites. [file MOL2-17-2147-s013.tif]

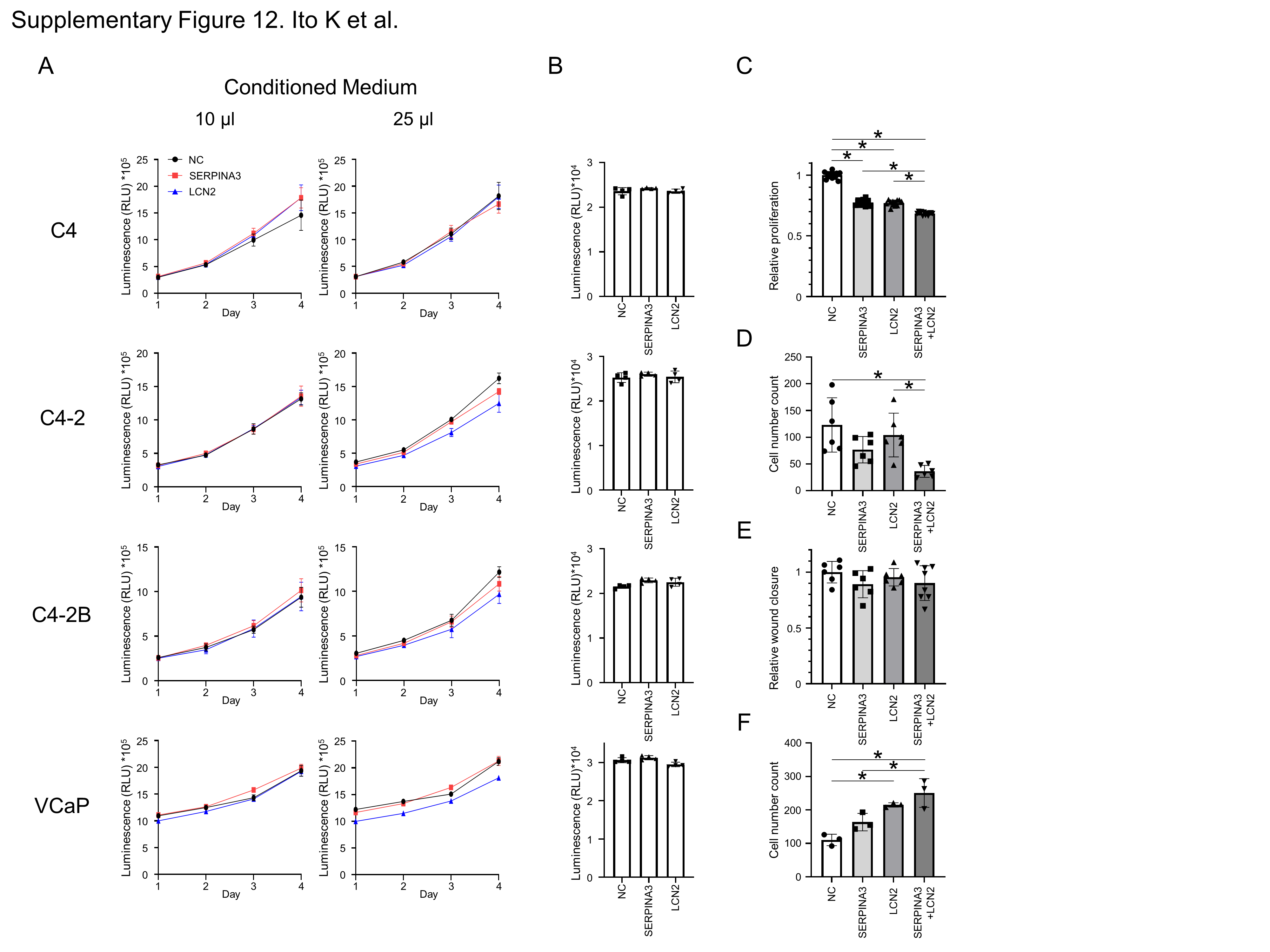

Supplement: Supplementary file 12 — Fig. S12. SERPINA3 and LCN2 effects on BPCa. (A) Luminescent cell viability assays of BPCa cells (C4, C4‐2, C4‐2B, and VCaP) with 10 and 25 μl/well of SERPINA3‐ or LCN2‐conditioned medium from HEK293T cells. n = 4, *P<0.05. (B) The relative caspase 3/7 activity in BPCa cells (C4, C4‐2, C4‐2B, and VCaP) treated with SERPINA3‐ or LCN2‐conditioned medium from HEK293T cells. Caspase 3/7 activity was measured after 24 hours of treatment. n = 4, *P<0.05. (C) C4‐2B cell lines (C4‐2B, C4‐2B‐SERPINA3, C4‐2B‐LCN2, and C4‐2B‐SERPINA3‐LCN2) proliferation measured by luminescent cell viability assays. n = 12, *P<0.05. (D) Invasion assay of C4‐2B cell line cultured in collagen‐coated Boyden chambers. n = 6, *P<0.05. (E) Wound healing assay of C4‐2B cell lines. n = 6, *P<0.05. (F) Soft agar colony formation assay of C4‐2B cell lines. n = 3, *P<0.05. [file MOL2-17-2147-s015.tif]

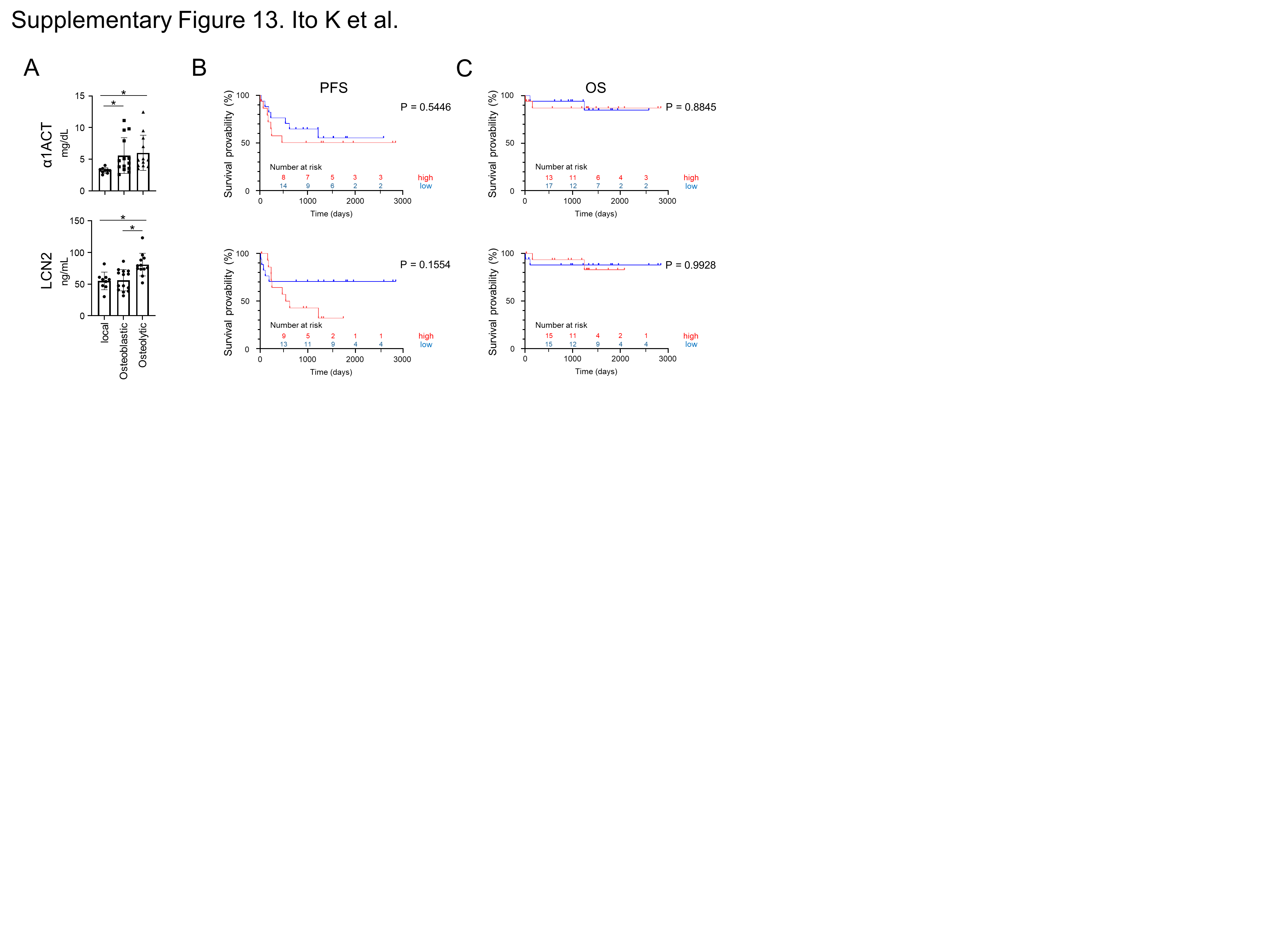

Supplement: Supplementary file 13 — Fig. S13. α1 ACT and LCN2 plasma concentration in PCa patients. (A) α1ACT and LCN2 concentrations in PCa patients without bone metastasis, with osteoblastic bone metastasis (OB) and with osteolytic bone metastasis (OL) from NCC patient plasma examined by an ELISA kit. *P<0.05 (B and C) Kaplan–Meier plots with SERPINA3 and LCN2 protein levels from NCC prostate cancer patient plasma using PFS (B) and overall survival (C). No significant correlation was found between plasma SERPINA3 or LCN2 concentrations and PFS or OS. [file MOL2-17-2147-s006.tif]

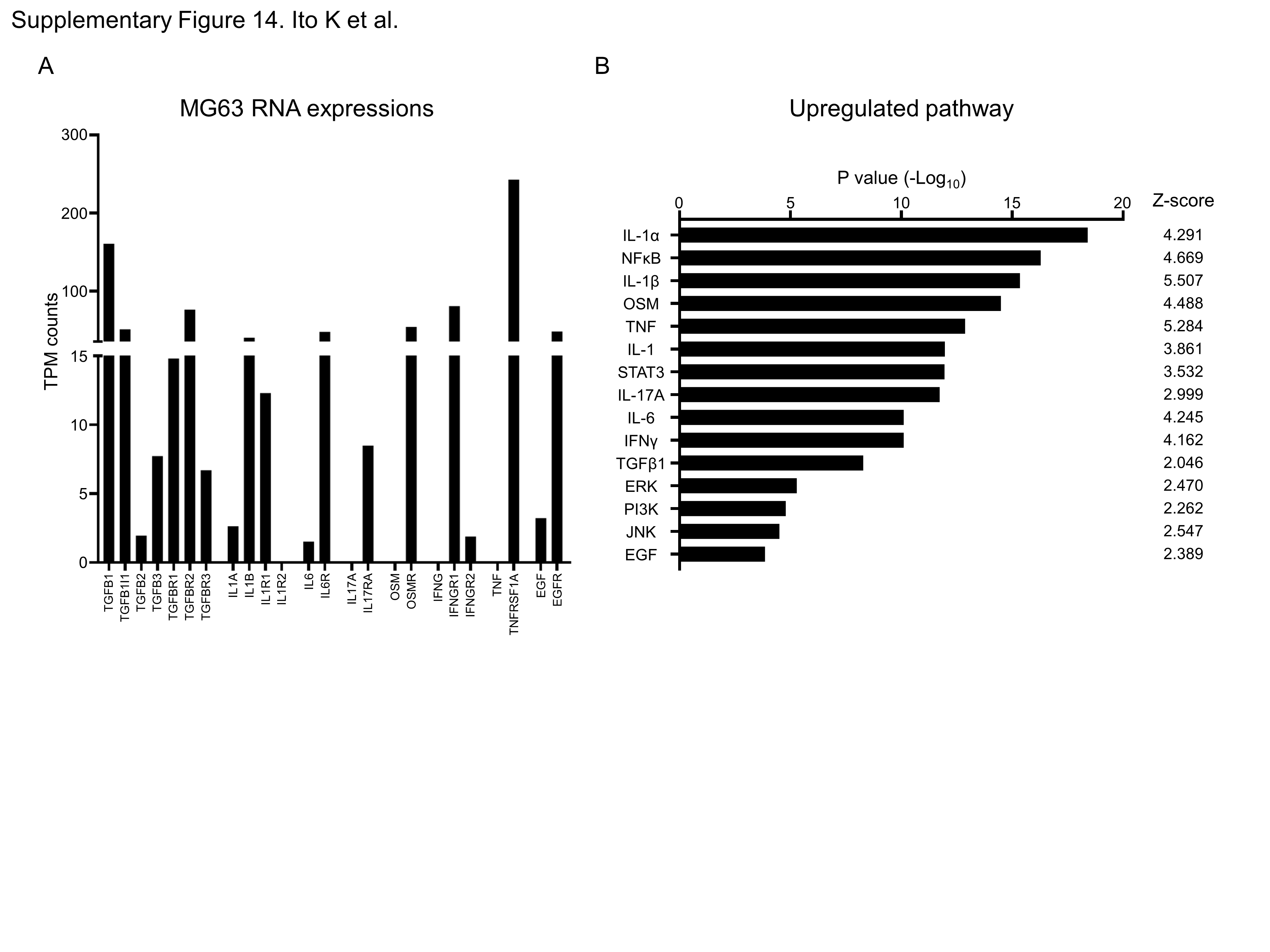

Supplement: Supplementary file 14 — Fig. S14. Upstream mediator candidates of SERPINA3 and LCN2. (A) TPM counts of MG63 cells RNA expression from RNA‐seq data in GEO database (GSE208277). (B) Significantly upregulated pathways in C4‐2B cells by horizontal co‐culturing with MG63 cells (C4‐2B co‐cultured with MG63 vs. C4‐2B). Ingenuity Pathway Analysis (IPA, https://www.digital‐biology.co.jp/allianced/products/ipa/) was performed to identify upstream regulators. [file MOL2-17-2147-s001.tif]
