# Supplementary material for: Osteoblast‐derived extracellular vesicles exert osteoblastic and tumor‐suppressive functions via SERPINA3 and LCN2 in prostate cancer
Source: Mol Oncol. 2023 Aug 4;17(10):2147–67. doi: 10.1002/1878-0261.13484 (PMC10552899; doi:10.1002/1878-0261.13484)
Supplement: Supplementary file 15 — Table S1. Primer sequences for qRT‐PCR. [file MOL2-17-2147-s003.docx]

| **Supplementary Table 1. Primer sequences for qRT-PCR.** | | |
| --- | --- | --- |
|  |  |  |
| **Race** | **Name** | **Sequence** |
| human | RANKL_Forward | ACCAGCATCAAAATCCCAAG |
| human | RANKL_Reverse | TAAGGAGGGGTTGGAGACCT |
| human | ALPL_Forward | CCTCCTCGGAAGACACTCTG |
| human | ALPL_Reverse | GCAGTGAAGGGCTTCTTGTC |
| human | BGLAP_Forward | GTGCAGAGTCCAGCAAAGGT |
| human | BGLAP_Reverse | TCAGCCAACTCGTCACAGTC |
| human | SPP1_Forward | TTGCAGTGATTTGCTTTTGC |
| human | SPP1_Reverse | GCCACAGCATCTGGGTATTT |
| human | SP7_Forward | TAATGGGCTCCTTTCACCTG |
| human | SP7_Reverse | CACTGGGCAGACAGTCAGAA |
| human | RUNX2_Forward | TTACTTACACCCCGCCAGTC |
| human | RUNX2_Reverse | TATGGAGTGCTGCTGGTCTG |
| human | LCN2_Forward | CCACCTCAGACCTGATCCCA |
| human | LCN2_Reverse | CCCCTGGAATTGGTT GTCCTG |
| human | SERPINA3_Forward | AAGGACCTTGACTCGCAGAC |
| human | SERPINA3_Reverse | CATGGGCACCATTACCCACT |
| human | ACTB_Forward | TAATGTCACGCACGATTTCCC |
| human | ACTB_Reverse | TCACCGAGCGCGGCT |
| mouse | B2m_Forward | ATGGGAAGCCGAACATACTG |
| mouse | B2m_Reverse | CAGTCTCAGTGGGGGTGAAT |
| mouse | Rank_Forward | TGCAGCTCAACAAGGATACG |
| mouse | Rank_Reverse | GTGCAGTTGGTCCAAGGTTT |
| mouse | Prdm1_Forward | ACACCGGGACTCCTACTCCT |
| mouse | Prdm1_Reverse | GTACGGTGGCAACAGGAACT |
| mouse | Nfatc1_Forward | TCATCCTGTCCAACACCAAA |
| mouse | Nfatc1_Reverse | TCACCCTGGTGTTCTTCCTC |
| mouse | Mitf_Forward | AACCGACAGAAGAAGCTGGA |
| mouse | Mitf_Reverse | TGATGATCCGATTCACCAGA |
| mouse | Trap_Forward | CAGCAGCCAAGGAGGACTAC |
| mouse | Trap_Reverse | ACATAGCCCACACCGTTCTC |
| mouse | Ctsk_Forward | TCTCTCGGCGTTTAATTTGG |
| mouse | Ctsk_Reverse | AAGTGGTTCATGGCCAGTTC |
| mouse | Dc-stamp_Forward | AAAACCCTTGGGCTGTTCTT |
| mouse | Dc-stamp_Reverse | AATCATGGACGACTCCTTGG |
| mouse | Spp1_Forward | TGCACCCAGATCCTATAGCC |
| mouse | Spp1_Reverse | CTCCATCGTCATCATCATCG |
| mouse | Bglap_Forward | GAACAGACAAGTCCCACACAGC |
| mouse | Bglap_Reverse | TCAGCAGAGTGAGCAGAAAGAT |
| mouse | Alpl_Forward | AACCCAGACACAAGCATTCC |
| mouse | Alpl_Reverse | GCCTTTGAGGTTTTTGGTCA |
| mouse | Col1a1_Forward | GCAAGAGGCGAGAGAGGTTT |
| mouse | Col1a1_Reverse | GACCACGGGCACCATCTTTA |
| mouse | Ibsp_Forward | AACAATCCGTGCCACTCACT |
| mouse | Ibsp_Reverse | GGCCGGTACTTAAAGACCCC |
