# Supplementary material for: Osteoblast‐derived extracellular vesicles exert osteoblastic and tumor‐suppressive functions via SERPINA3 and LCN2 in prostate cancer
Source: Mol Oncol. 2023 Aug 4;17(10):2147–67. doi: 10.1002/1878-0261.13484 (PMC10552899; doi:10.1002/1878-0261.13484)
Supplement: Supplementary file 16 — Table S2. Baseline demographics of patients from the National Cancer Center, Japan. [file MOL2-17-2147-s010.docx]

| **Supplementary Table 2. Baseline demographics of patients from the National Cancer Center, Japan.** | | | | | | |
| --- | --- | --- | --- | --- | --- | --- |
|  |  |  |  |  |  |  |
|  |  | local PCa | osteoblastic bone metastasis | osteolytic bone metastasis | P value | |
|  |  |  |  |  | local VS metastasis | lytic VS blastic |
| n |  | 21 | 19 | 12 |  |  |
| age |  | 66.5 (39-79) | 69.8 (50-84) | 68.5 (55-81) | 0.2983 | 0.7051 |
| PSA |  | 13.71 (4.927-117.77) | 1237 (18.791-14199) | 1774 (95.462-6478.9) | 0.0232 | 0.6105 |
| Gleason Score (%) | 3+3 | 0 (0) | 0 (0) | 0 (0) | 0.0023 | 0.279 |
|  | 3+4 | 6 (40) | 1 (5.6) | 0 (0) |  |  |
|  | 4+3 | 5 (33.3) | 5 (27.8) | 1 (10) |  |  |
|  | 4+4 | 1 (6.7) | 2 (11.1) | 2 (20) |  |  |
|  | 4+5 | 2 (13.3) | 6 (33.3) | 4 (40) |  |  |
|  | 5+4 | 1 (6.7) | 4 (22.2) | 3 (30) |  |  |
|  | 5+5 | 0 (0) | 0 (0) | 0 (0) |  |  |
| NSE |  | 10.51 (8.6-13.6) | 15.88 (8.1-45.6) | 17.60 (8-56.2) | 0.1735 | 0.7249 |
| ALP |  | 270 (91-448) | 585.7 (72-2319) | 716.5 (227-2530) | 0.0080 | 0.5619 |
| T score | ≤3 | 21 | 18 | 12 | 0.9999 | 0.9999 |
|  | 4 | 0 | 1 | 0 |  |  |
| N score | 0 | 15 | 9 | 2 | 0.0001 | 0.1255 |
|  | 1 | 0 | 9 | 9 |  |  |
| metastasis | bone | 0 | 19 | 12 | 0.0001 | 0.9999 |
|  | visceral | 0 | 4 | 4 | 0.0155 | 0.6757 |
| Initial treatment | Surgery | 9 | 0 | 0 | 0.0001 | 0.9999 |
|  | Radiation | 5 | 0 | 0 |  |  |
|  | ADT | 4 | 19 | 12 |  |  |
